# Prevalence of early-onset neonatal infection among newborns of mothers with bacterial infection or colonization: a systematic review and meta-analysis

**DOI:** 10.1186/s12879-015-0813-3

**Published:** 2015-03-07

**Authors:** Grace J Chan, Anne CC Lee, Abdullah H Baqui, Jingwen Tan, Robert E Black

**Affiliations:** 1 grid.2515.30000000403788438Boston Children’s Hospital, Boston, MA 02115 USA; 2 grid.38142.3c000000041936754XHarvard T.H. Chan School of Public Health, Boston, MA USA; 3 grid.62560.370000000403788294Brigham and Women’s Hospital, Boston, MA USA; 4 grid.21107.350000000121719311Johns Hopkins School of Public Health, Baltimore, MD 21205 USA

**Keywords:** Early-onset, Neonatal infection, Maternal infection, Systematic review, Meta-analysis

## Abstract

**Background:**

Although neonatal infections cause a significant proportion of deaths in the first week of life, little is known about the burden of neonatal disease originating from maternal infection or colonization globally. This paper describes the prevalence of vertical transmission – the percentage of newborns with neonatal infection among newborns exposed to maternal infection.

**Methods:**

We searched Pubmed, Embase, Scopus, Web of Science, Cochrane Library, and WHO Regional Databases for studies of maternal infection, vertical transmission, and neonatal infection. Studies that measured prevalence of bacterial vertical transmission were included. Random effects meta-analyses were used to pool data to calculate prevalence estimates of vertical transmission.

**Results:**

122 studies met the inclusion criteria. Only seven studies (5.7%) were from very high neonatal mortality settings. Considerable heterogeneity existed between studies given the various definitions of infection (lab-confirmed, clinical signs), colonization, and risk factors of infection. The prevalence of early onset neonatal lab-confirmed infection among newborns of mothers with lab-confirmed infection was 17.2% (95%CI 6.5-27.9). The prevalence of neonatal lab-confirmed infection among newborns of colonized mothers was 0% (95% CI 0.0-0.0). The prevalence of neonatal surface colonization among newborns of colonized mothers ranged from 30.9-45.5% depending on the organism. The prevalence of neonatal lab-confirmed infection among newborns of mothers with risk factors (premature rupture of membranes, preterm premature rupture of membranes, prolonged rupture of membranes) ranged from 2.9-19.2% depending on the risk factor.

**Conclusions:**

The prevalence of early-onset neonatal infection is high among newborns of mothers with infection or risk factors for infection. More high quality studies are needed particularly in high neonatal mortality settings to accurately estimate the prevalence of early-onset infection among newborns at risk.

**Electronic supplementary material:**

The online version of this article (doi:10.1186/s12879-015-0813-3) contains supplementary material, which is available to authorized users.

## Background

Neonatal infections account for a significant proportion of neonatal deaths in the first week of life [1]. In sub-Saharan Africa, south Asia, and Latin America where neonatal infections are most prevalent, the case fatality risk associated with possible severe bacterial infections in the first month of life is 9.8% [2]. Infections are one of the three major causes of neonatal mortality and account for approximately a quarter of newborn deaths in the first month of life [3]. Neonatal infections are acquired horizontally (from the environment) or vertically (from mother). Not much is known about the routes of transmission globally where different environments and risk factors may affect paths of transmission. In resource-rich settings, interventions such as intrapartum antibiotic prophylaxis for high risk women has been effective in reducing the incidence of early-onset neonatal sepsis. In contrast, these interventions are rare or absent in resource-poor settings, which have the highest rates of neonatal mortality. To develop research priorities and strategies for prevention, we need to better understand the prevalence of neonatal infections that are maternally acquired. In this systematic review and meta-analysis, we estimate the prevalence of early-onset neonatal infection in cases where the pregnant woman was infected or colonized with bacterial pathogens (hereafter referred to “vertical transmission”) to better understand the global rates of vertical transmission. We used a relaxed inclusion criteria to include studies that at minimum measured maternal infections and neonatal infections without necessarily having the comparison group of women without maternal infections.

## Methods

### Definitions and classification

Although laboratory confirmed infections are considered the gold standard measure of infection, the limited number of studies in African, eastern Mediterranean, and southeast Asian regions with laboratory capabilities would underestimate the prevalence of neonatal sepsis among pregnant women who were infected or colonized. Rather than restricting to only lab-confirmed definitions, we also included clinical signs, colonization, and risk factors for infection (maternal only) to best estimate the prevalence of vertical transmission. We specified these definitions, our methods of analysis, and our inclusion criteria in a protocol *a priori*.

We defined our exposure, maternal infection or colonization during labor, in three categories:

(i) Maternal Infection
  Laboratory confirmed bacterial infection (hereafter referred to as “lab”): culture or PCR confirmed bacteremia, amnionitis, urinary tract infections, chorioamnionitis;
  Clinical signs of infection (hereafter referred to as “signs”): intrapartum maternal fever, uterine tenderness, maternal tachycardia, malodorous vaginal discharge, elevated white cell count, elevated c-reactive protein, physician diagnosis of clinical chorioamnionitis using a combination of the above signs, or clinical infection undefined.
(ii) Maternal Colonization: positive reproductive tract/genital bacterial cultures without signs or symptoms of infection.
(iii) Risk factors for infection: premature rupture of membranes (PROM - rupture of membranes prior to onset of labor ≥ 37 weeks gestation), preterm premature rupture of membranes (PPROM - rupture of membranes prior to onset of labor < 37 weeks gestation), and prolonged rupture of membranes (duration of rupture of membranes [ROM] ≥ 18-24 hours or undefined).

The outcome, early-onset neonatal infection or colonization during the first seven days of life, was defined in two categories:

(i) Neonatal Infection
  Laboratory confirmed bacterial infection (“lab”): bacteremia, meningitis, urinary tract infection (positive culture of blood, cerebral spinal fluid, or urine);
  Clinical signs of infection (“signs”): pneumonia, fever, hypothermia, respiratory distress, bradycardia, tachycardia, irritability, lethargy, hypotonia, seizures, poor feeding, oxygen requirement, increased frequency of apnea, poor capillary refill, metabolic acidosis, elevated white cell count, high immature to total neutrophil ratio, elevated c-reactive protein, or physician diagnosis of clinical sepsis using a combination of the above signs;
  Laboratory or clinical infection (hereafter referred to as “lab/lab&signs”): a combination of either laboratory confirmed infection or clinical signs of infection, or undefined.
(ii) Newborn Colonization: positive ear canal, umbilical, axilla, or anal cultures without signs or symptoms of infection.

We use the term “maternal exposure” as an overarching description of exposures and “neonatal outcome” to describe the outcomes.

### Search strategy and section criteria

We searched Pubmed (Medline), Embase, Scopus, Web of Science, the Cochrane Library, and the World Health Organization (WHO) Regional Databases. A comprehensive search strategy was developed with three concepts: maternal infection, vertical transmission, and neonatal infection (Additional file 1: Table S1). We performed final searches of databases on February 20, 2012 with no date restrictions. We downloaded and reviewed articles using EndNote (version X4). Hand-searches through the reference lists of screened articles and published systematic reviews did not produce any additional articles. Source articles included publications, abstracts, and conference proceedings available in the public domain.

We included studies of any design that measured the prevalence or incidence (assumed to be period prevalence) of bacterial vertical transmission, or contained raw data to calculate these measures, even if vertical transmission was not the main aim of the study. To be included, studies needed to be original full-text articles, provide a measure of maternal exposures and a measure of neonatal outcomes, and be written in English. We excluded studies if: the sample size was less than ten; all subjects (pregnant women) received antibiotics or steroids; or data related to nonbacterial infections, tetanus infections, or sexually transmitted infections such as chlamydia and syphilis, which have different mechanisms of transmission.

### Screening and data abstraction

Two reviewers independently screened titles, abstracts, and articles using predetermined selection criteria and standardized data abstraction forms. Two data abstractors independently gathered data from included studies to assess risk of bias, classify exposures and outcomes, determine the number of newborns in each exposure-outcome category, and calculate a prevalence measure. One reviewer abstracted study characteristics data. To improve data quality, a second reviewer abstracted study characteristics for a random 10% of the studies. At each stage, the reviewers compared their results and resolved disagreements by reaching a consensus. For articles missing information critical to our analysis, we contacted the authors to request the missing data.

We obtained basic data on author, country, study design, sample size, and study setting: (1) health facility, multi-center, or community-based, and (2) urban versus rural. The studies provided limited data on intrapartum antibiotic use; we categorized studies based on whether they had no intrapartum antibiotic use, some antibiotic use, or unknown if the study did not report antibiotic use. We defined our outcome, early-onset neonatal infection, to the first seven days of life. Studies that used the term “early-onset neonatal sepsis” but did not specify timing (i.e. seven days or three days) were also included. We also included studies that examined only high risk populations such as preterm labor, PROM, PPROM, and prolonged rupture of membranes. To assess for variations by region, we grouped studies by World Health Organization region, 2010 World Bank gross national income per capita in US dollars (low $1005 or less, lower-middle $1006-3975, upper-middle $3976-12 275, high income $12 276 or more), and 2009 UNICEF neonatal mortality rates (very low <5 deaths per 1000 live births, low 5-14 deaths/1000, high 15-27 deaths/1000, and very high more than 27 deaths/1000) [4-6].

Two independent reviewers assessed the methodological quality of included studies, examining selection methods, missing data, loss-to-follow-up, misclassification or measurement errors of exposures or outcomes, and confounding bias. Biases in selection and misclassification would have the largest effect on prevalence results. Studies were given an overall rating of low risk of bias if both selection and misclassification biases were at low risk. Studies that were high risk for either selection or misclassification bias or both were rated as high risk of bias. Studies that did not meet the low or high risk criteria were rated as having unclear risk of bias.

### Statistical analysis

We used random-effects meta-analyses to calculate weighted mean estimates across studies and the 95% CI for the point prevalence of vertical transmission (Stata v12). If raw data were not available, we used the reported vertical transmission prevalence and calculated the standard error SE = √ [p(1 - p)/n]. If there were two or more studies included in the meta-analysis, we assessed measures of heterogeneity with I^2^ statistics. For each combination of maternal exposure and neonatal outcome, we calculated a pooled estimate of the prevalences. Because of the substantial heterogeneity across all combinations of maternal exposures and neonatal outcomes, we did not calculate an overall pooled estimate of the prevalences.

The studies we examined used numerous combinations of maternal exposure and neonatal outcome. This paper presents the following combinations:

i) maternal lab confirmed infection and neonatal lab confirmed infection (lab/lab);
ii) maternal lab confirmed infection and neonatal clinical signs of infection (lab/signs);
iii) maternal lab confirmed infection and neonatal lab or clinical infection (lab/lab&signs);
iv) maternal clinical signs of infection and neonatal lab confirmed infection (signs/lab);
v) maternal clinical signs of infection and neonatal clinical signs of infection (signs/signs);
vi) maternal clinical signs of infection and neonatal lab or clinical infection (signs/lab&signs);
vii) maternal colonization and neonatal lab confirmed infection (colonization/lab);
viii) maternal colonization and neonatal clinical signs of infection (colonization/signs);
ix) maternal colonization and neonatal lab or clinical infection (colonization/lab&signs);
x) maternal colonization and neonatal colonization (colonization/colonization);
xi) maternal risk factor and neonatal lab confirmed infection (risk/lab);
xii) maternal risk factor and neonatal clinical signs of infection (risk/signs).

The four forest plots presented in this paper estimate the vertical transmission point prevalence, 95% CI, and relative weights for four different groupings of these combinations: (i-vi) maternal infection and neonatal infection; (vii-ix) maternal colonization and neonatal infection; (x) maternal colonization and neonatal colonization; and (xi-xii) maternal risk factors and neonatal infections.

We originally planned for subgroup analyses by region, gross national income per capita in US dollars stratum, intrapartum antibiotic use, and neonatal mortality rate stratum. However, given the scarcity of data in the lower income and higher mortality rates countries, we were only able to describe the distribution of studies by region, income, and neonatal mortality rate. For the maternal colonization and neonatal colonization analysis, we examined pathogen-specific subgroups with *Staphylococcus aureus,* non-group B *Streptococcus* species, Group B streptococcus, *Klebsiella pneumoniae*, *Escherichia coli*, *Ureaplasma* species, *Mycoplasma hominis*, and multiple organisms. In our sensitivity analyses, we repeated meta-analyses excluding studies with (i) some or unknown intrapartum antibiotics use to understand the natural history of vertical transmission in settings of most LIC where intrapartum antibiotics are not available and (ii) high risk of bias. We planned these subgroup and sensitivity analyses *a priori*.

### Role of the funding source

The funding source had no role in the study design, data collection, analysis, interpretation, or writing of the report. The corresponding author had full access to all the data in the study and had final responsibility for the decision to submit the publication.

## Results

**Figure 1:**
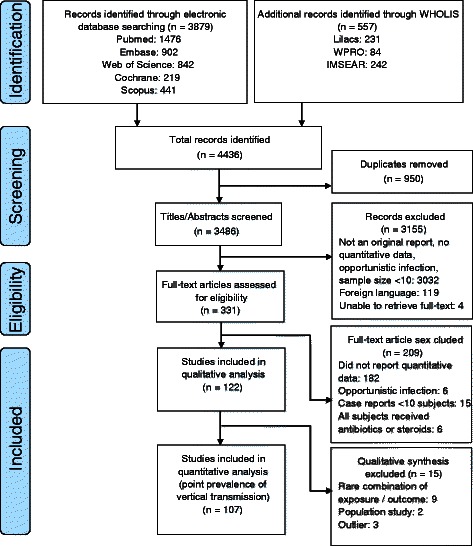
**Flow diagram of study selection.**

Our search identified 4436 articles of which 3486 were unique records. We reviewed 331 full-text articles. Eighteen authors were contacted regarding missing data and provided with a sample 2×2 table to complete. One [7] out of the four authors who responded provided usable data. Data from 122 studies met the inclusion criteria. Our qualitative analysis included all 122 studies; our quantitative meta-analysis included 107 of them (Figure 1, Additional file 2: Table S2). The majority of studies used cohort designs (n = 117, 95.9%) set in single health facilities (n = 94, 77.0%). In 75 studies (61.5%), researchers measured early-onset neonatal infection during the first seven days of life. Twenty-eight studies (23.0%) had data on women who did not use intrapartum antibiotics (either the study cohort did not use antibiotics, the study excluded women who received antibiotics, or data were abstracted from the placebo arm of an intervention trial); 51 studies (41.8%) reported some antibiotic use in a subset of women for prophylaxis or treatment; and 43 studies (35.2%) did not specify whether antibiotics were used. A sensitivity analysis around studies without or unknown use of antibiotics was limited by the data available and only possible in the subgroup maternal colonization and neonatal infection.

Thirty eight of the studies (31.1%) restricted their enrolment to a specific subset of women (preterm labor, PROM, PPROM, prolonged rupture), while a majority of studies (n = 80, 65.6%) examined all pregnant women. Four studies (3.3%) did not report the inclusion or exclusion criteria. Most studies (n = 101, 82.8%) occurred in the Americas or Europe, ten studies (8.2%) were in the western Pacific, five studies (4.1%) were in southeast Asia, three studies (2.5%) were in the eastern Mediterranean region, and three studies (2.5%) were in Africa. Most studies (n = 104, 85.2%) were in high income and low mortality settings (Table 1). Table 1 describes the number of studies and study characteristics in each meta-analysis. A study could report more than one maternal condition but was used only once in each meta-analysis (Additional file 3: Table S3, Additional file 4: Table S4).

**Table 1:** **Characteristics of included studies**

	**Total (all studies)**	**Maternal infections and neonatal infections**	**Maternal colonization and neonatal infections**	**Maternal colonization and neonatal colonization**	**Maternal risk factors and neonatal infections**
Total number of studies (qualitative and meta-analysis)*	122	37	37	39	27
Number of studies in the meta-analysis*	107	32	36	38	27
Study sample size, median (25th, 75th percentile)	337 (IQR 144-1413)	146 (IQR 53-524)	937 (IQR 201-2040)	800 (IQR 317-1457)	225 (IQR 94-1280)
**Study type**					
Cohort (including RCTs)	117 (95.9%)	34 (91.9%)	36 (97.3%)	39 (100%)	26 (96.3%)
Nested case-control	2 (1.6%)	1 (2.7%)	1 (2.7%)	--	1 (3.7%)
Population surveillance	3 (2.5%)	2 (5.4%)	--	--	--
**Location**					
Health facility	94 (77.0%)	28 (75.7%)	28 (75.7%)	31 (79.5%)	21 (77.8%)
Multi-center	24 (19.7%)	7 (18.9%)	7 (18.9%)	7 (18.0%)	6 (22.2%)
Unknown or not clear	4 (3.3%)	2 (5.4%)	2 (5.4%)	1 (2.6%)	--
**Urban or rural**					
Urban or periurban	99 (81.2%)	30 (81.1%)	28 (75.7%)	33 (84.6%)	22 (81.5%)
Mixed	2 (1.6%)	1 (2.7%)	--	--	--
Unknown or not clear	21 (17.2%)	6 (16.2%)	9 (24.3%)	6 (15.4%)	5 (18.5%)
**Timing of early-onset sepsis**					
First seven days of life	75 (61.5%)	18 (48.6%)	24 (64.9%)	31 (79.5%)	15 (55.6%)
Not reported or unclear	47 (38.5%)	19 (51.4%)	13 (35.1%)	8 (20.5%)	12 (44.4)
**Antibiotic use**					
No intrapartum antibiotic use	28 (23.0%)	4 (10.8%)	10 (27.0%)	10 (25.6%)	9 (33.3%)
Some intrapartum antibiotic use	51 (41.8%)	21 (56.8%)	17 (46.0%)	13 (33.3%)	11 (40.7%)
Unknown or not clear	43 (35.2%)	12 (32.4%)	10 (27.0%)	16 (41.0%)	7 (25.9%)
**High risk population**					
Preterm	8 (6.6%)	7 (18.9%)	--	--	3 (11.1%)
PROM	5 (4.1%)	2 (5.4%)	1 (2.7%)	--	4 (14.8%)
PPROM	17 (13.9%)	7 (18.9%)	2 (5.4%)	--	8 (29.6%)
Prolonged rupture of membranes	1 (0.8%)	--	--	--	1 (3.7%)
Preterm or PROM	5 (4.1%)	2 (5.4%)	2 (5.4%)	1 (2.6%)	3 (11.1%)
Preterm or PPROM	2 (1.6%)	--	--	--	2 (7.4%)
None, all women included	80 (65.6%)	17 (45.0%)	31 (83.8%)	36 (92.3%)	6 (22.2%)
Other or unclear	4 (3.3%)	2 (5.4%)	1 (2.7%)	2 (5.1%)	--
**WHO Region**					
Africa	3 (2.5%)	--	--	2 (5.1%)	1 (3.7%)
Americans	52 (42.6%)	23 (62.2%)	16 (43.2%)	6 (15.4%)	16 (59.3%)
Eastern Mediterranean	3 (2.5%)	--	1 (2.7%)	3 (7.7%)	--
Europe	49 (40.2%)	8 (21.6%)	11 (29.7%)	23 (59.0%)	8 (29.6%)
Southeast Asia	5 (4.1%)	(5.4%)	3 (8.1%)	2 (5.1%)	1 (3.7%)
Western Pacific	10 (8.2%)	4 (10.8%)	6 (16.2%)	3 (7.7%)	1 (3.7%)
**Neonatal mortality range**					
Very low mortality <5 per 1000 live births	104 (85.2%)	34 (91.9%)	32 (86.5%)	28 (71.8%)	23 (85.2%)
Low mortality 5-14	8 (6.6%)	1 (2.7%)	--	6 (15.4%)	2 (7.4%)
High mortality 15-27	3 (2.5%)	--	2 (5.4%)	2 (5.1%)	--
Very high mortality >27	7 (5.7%)	2 (5.4%)	3 (8.1%)	3 (7.7%)	2 (7.4%)
**Income range**					
High income (≥12276) per capita in USD	104 (85.3%)	34 (91.9%)	32 (86.5%)	29 (74.4%)	22 (81.5%)
Upper middle income (3976-12275)	11 (9.0%)	1 (2.7%)	1 (2.7%)	7 (18.0%)	3 (11.1%)
Lower middle income (1006-3975)	6 (4.9%)	2 (5.4%)	4 (10.8%)	3 (7.7%)	1 (3.7%)
Low income (≤1005)	1 (0.8%)	--	--	--	1 (3.7%)

*Studies could be included in more than one meta-analysis (Maternal infections and neonatal infections, Maternal colonization and neonatal infections, Maternal colonization and neonatal colonization, and Maternal risk factors and neonatal infections).

Using available data from this pool of studies, we calculated the following median prevalence of exposures and outcomes: maternal lab-confirmed infection (17 studies, median prevalence 24.0%, interquartile range [IQR] 10.0-28.3), maternal clinical signs of infection (8 studies, median prevalence 11.6%, IQR 2.7-15.6), maternal colonization (60 studies, median prevalence 16.3%, IQR 7.9-23.5), neonatal lab-confirmed infection (37 studies, median prevalence 1.5%, IQR 0.1-7.1), neonatal clinical signs of infection (8 studies, median prevalence 11.3%, IQR 4.5-24.9), neonatal lab-confirmed or clinical signs of infection (8 studies, median prevalence 9.3%, IQR 6.0-16.8), and neonatal colonization (31 studies, median prevalence 7.3%, IQR 3.8-17.3). Ten studies defined maternal exposures or neonatal outcomes in categories that were too heterogeneous to place in a meta-analysis [8-17].

### Regional

Available data on laboratory cultures, clinical signs, colonization status, and risk factors varied by region. The Americas, Europe and western Pacific regions had studies that examined maternal lab-confirmed, clinical signs, colonization, and risk factors. None of the studies in Africa, the eastern Mediterranean, and southeast Asia provided lab-confirmed maternal infection data, and no studies in Africa, the eastern Mediterranean, and the western Pacific provided clinical signs data. We were able to find studies in every region with maternal colonization data and with neonatal lab confirmed infection and colonization data, with the majority in the Americas and Europe. There were no studies of neonatal clinical infection from Africa or the eastern Mediterranean.

### Risk of bias

Among the 122 included studies, 10 (8.2%) studies were rated overall as low risk; 57 (46.7%) were rated as unclear risk; and 55 (45.1%) were rated as high risk after considering selection and misclassification biases (Additional file 5: Figure S1). Eighteen (14.8%) studies had different eligibility criteria or baseline characteristics between the exposed and comparison groups. These studies were at high risk of selection bias. In assessing for misclassification bias, the majority of studies measured exposures or outcomes using reliable laboratory based culture methods. However, 24 (19.6%) studies determined the exposure from risk factors or clinical signs and 18 (14.7%) studies determined the outcome from clinical signs of sepsis.

### Meta-analyses

The meta-analysis results are presented by exposure outcome combination: (i) maternal infection and neonatal infection; (ii) maternal colonization and neonatal infection; (iii) maternal colonization and neonatal colonization; and (iv) maternal risk factors and neonatal clinical infection. The study characteristics and exposure/outcome definitions for individual study data are shown in the Additional file 2: Table S2 and Additional file 3: Table S3.

#### Maternal infection and neonatal infection

Thirty-eight studies reported data on maternal infections and neonatal infections. We excluded five studies from the meta-analysis: three studies that measured maternal urinary tract infection exposure, which reported zero to two percent vertical transmission rates, and two population surveillance studies that looked at only at rare infections such as *Listeria* species and *Bacteriodes* species infections [11,18-21].

**Figure 2:**
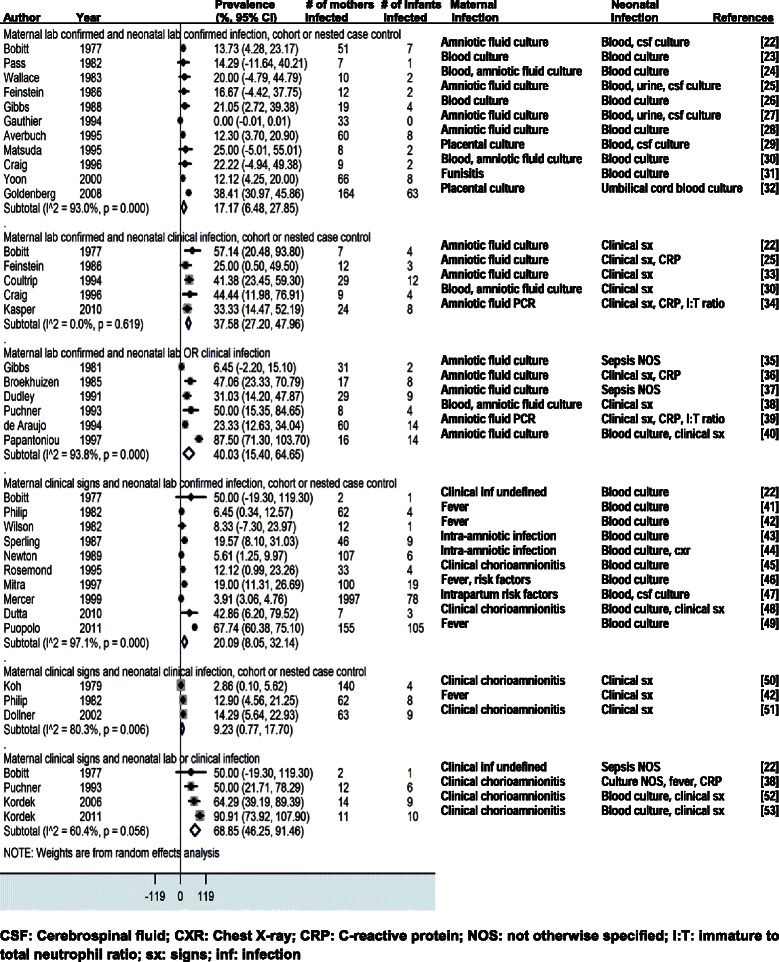
**Maternal infection and neonatal infection** [22-53].

In studies where mothers had lab-confirmed infection, 17.2% (95% CI 6.5-27.9) of their newborns were infected (“lab/lab”) (Figure 2). Of the 11 studies reporting lab-confirmed maternal infection in the “lab/lab” meta-analysis, four (36.4%) examined amniotic fluid cultures, two (18.2%) used blood cultures, two studied blood and/or amniotic fluid cultures, two tested placental swab cultures, and one (9.1%) detected funisitis by histologic examination of the umbilical cord. A sensitivity analysis excluding high risk for bias studies produced a slightly increased prevalence of 19.5% (95% CI 2.3-36.8).

Similarly, in studies where mothers were diagnosed with clinical signs of infection, 20.1% (95% CI 8.1-32.1) of their newborns had lab confirmed infection (“signs/lab”). Of the ten studies reporting maternal clinical signs of infection in the “signs/lab” analysis, four (40.0%) examined intrapartum fever, two (20.0%) clinical chorioamnionitis, two (20.0%) intra-amnionitic infection, one (10.0%) intrapartum risk factors, and one (10.0%) nonspecific clinical infection.

In studies where mothers had lab-confirmed infection, 37.6% (95% CI 27.2-48.0) of their newborns had clinical signs of infection (“lab/signs”). In studies where mothers had lab-confirmed infection, 40.0% (95% CI 15.4-64.7) of their newborns had lab or clinical signs of infection (“lab/lab&signs”). Sufficient data were not available for the sensitivity analysis separating studies by antibiotic use.

#### Maternal colonization and neonatal infection

**Figure 3:**
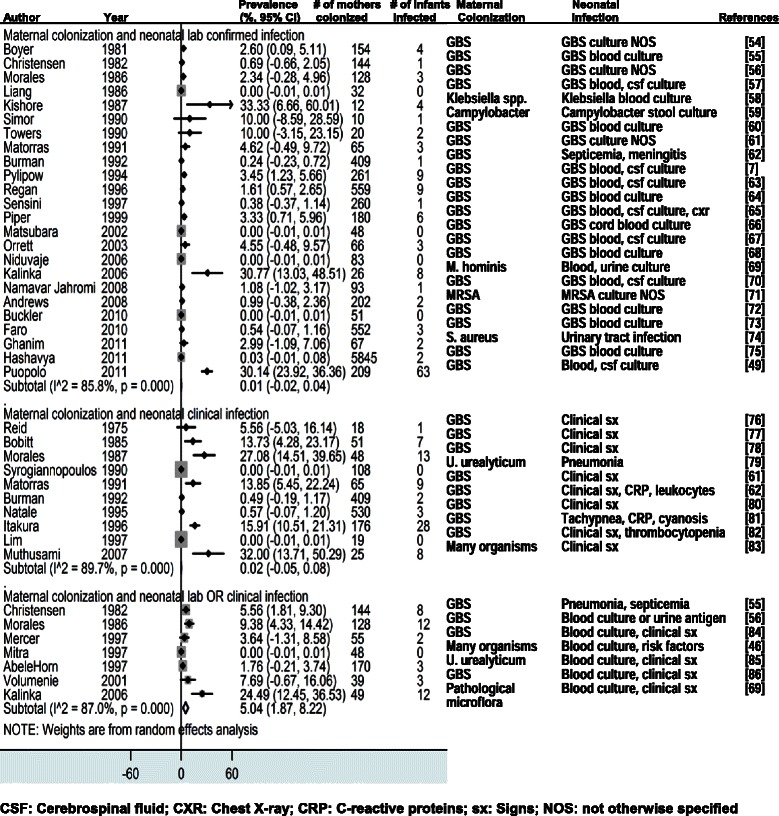
**Maternal colonization and neonatal infection** [54-86]**.**

Thirty-seven studies reported data on maternal colonization and neonatal infections. One study, Craig et al, was excluded from the meta-analysis as an outlier, as it reported that 80% of newborns of colonized mothers were infected [30]. In studies that measured neonatal infection with lab tests (“colonization/lab”), the prevalence of neonatal infection among newborns of colonized mothers was 0% (95% CI 0.0-0.0) (24 studies, median 2.0%, IQR 0.3-4.6) and 0% (95% CI -0.1-0.1) (10 studies, median 9.6%, IQR 1.8-9.4) in studies that measured neonatal infection with clinical signs (“colonization/signs”). In studies that used lab tests and collected clinical signs of neonatal infection (“colonization/lab&signs”), 5.0% (95% CI 1.9-8.2) (7 studies, median 5.6%, IQR 1.8-9.4) of colonized mothers had newborns with infection (Figure 3). A sensitivity analysis excluding studies with known or possible antibiotic use showed a slight increase in prevalence of neonatal infections in studies that tested lab outcomes (1.1%, 95% CI 0.2-2.0), studies that diagnosed clinical signs of neonatal infection (6.5%, 95% CI -6.5-19.5), and studies that collected clinical signs of infection and conducted labs tests (7.6%, 95% CI 1.4-13.8).

Most studies focused on maternal Group B Streptococcus (GBS) colonization. We conducted meta-analyses by region of studies that tested maternal GBS colonization and that measured neonatal infection with lab tests (“colonization/lab”). In the Americas, 3.2% (95% CI 1.8-4.7) (10 studies, median 3.0%, IQR 1.6-4.5) of GBS colonized mothers had newborns with infection. In Europe, 0.2% (95% CI -0.1-0.4) (4 studies, median 0.4%, IQR 0.2-0.7) of GBS colonized mothers had newborns with infection. In western Pacific region, 0.0% (95% CI 0.0-0.0) (2 studies, median 0.0%, IQR 0.0-0.0) of GBS colonized mothers had newborns with infection. We did not have sufficient data to conduct a sensitivity analysis excluding studies with some or unknown antibiotic use.

#### Maternal colonization and neonatal colonization

**Figure 4:**
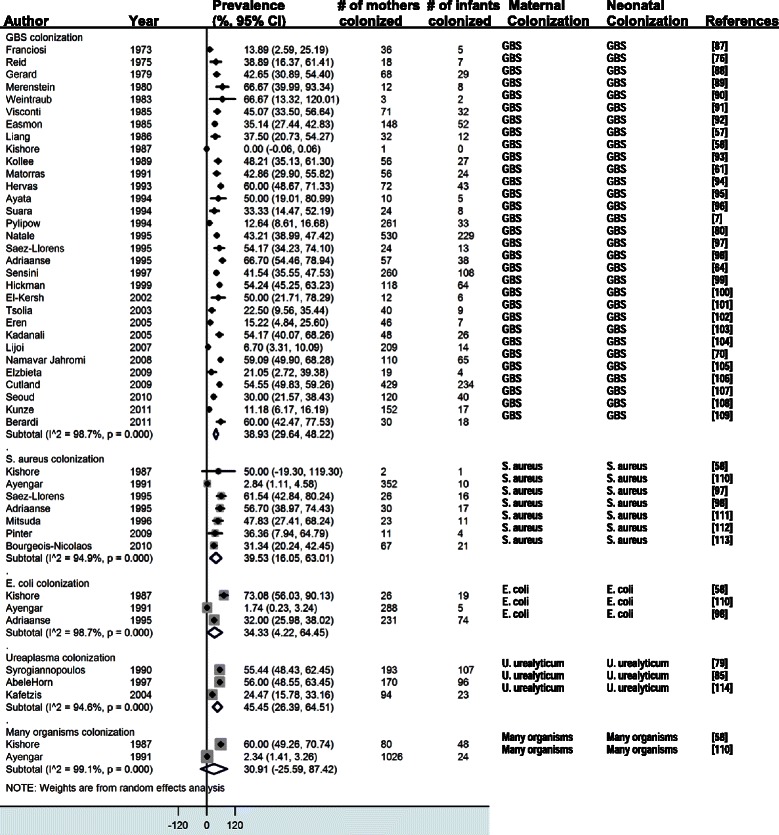
**Maternal colonization and neonatal colonization** [87-114]**.**

Thirty-nine studies reported data on maternal colonization and neonatal colonization (“colonization/colonization”). We present these results by pathogen-specific subgroups. In thirty-one studies where mothers were colonized with GBS, 38.9% (95% CI 29.6-48.2) of the newborns had surface GBS colonization. In seven studies where mothers were colonized with *Staphylococcus aureus*, 39.5% (95% CI 16.1-63.0) of the newborns had surface *S. aureus* colonization. In three studies where mothers were colonized with *Escherichia coli*, 34.3% (95% CI 4.2-64.5) of the newborns had *E. coli* colonization. In three studies where mothers were colonized with *Ureaplasma* species, 45.5% (95% CI 26.4-64.5) of the newborns were colonized with *Ureaplasma* species. Two studies did not differentiate clearly between organisms. In these studies, 30.9% (95% CI 25.6-87.4) of the newborns were colonized (Figure 4).

#### Maternal risk factors and neonatal infection

**Figure 5:**
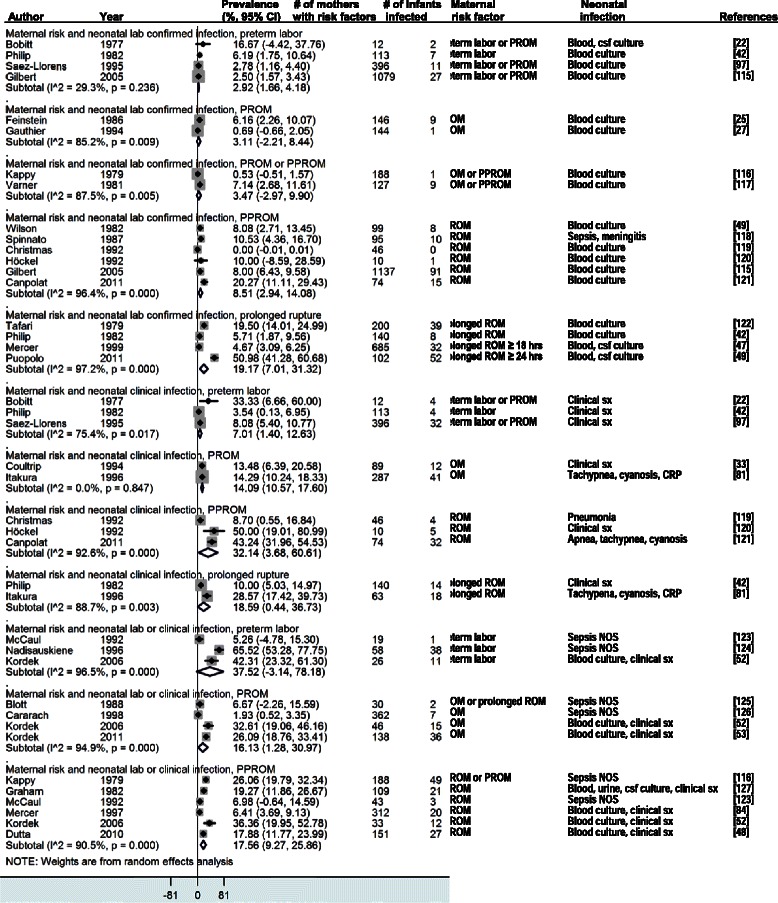
**Maternal risk factors and neonatal infection** [115-127]**.**

Twenty-seven studies presented data on maternal risk factors and neonatal infections. All were included in the meta-analysis. In studies where mothers had prolonged rupture of membranes, 19.2% (95% CI 7.0-31.3) of the newborns had positive lab cultures for infection. In studies where mothers had PPROM, 8.5% (95% CI 2.9-14.1) of the newborns had positive lab cultures for infection. In studies where mothers had PROM or PPROM, 3.5% (95% CI -3.0-9.9) of newborns had positive lab cultures for infection. In studies where mothers had PROM, 3.1% (95% CI -2.2-8.4) of the newborns had positive lab cultures. In studies where mothers experienced preterm labor, 2.9% (95% CI 1.7-4.2) of the newborns had positive lab cultures (Figure 5).

The prevalence of neonatal infection was higher in studies that measured neonatal clinical signs of infection (“risk/signs”). In studies where mothers had preterm labor, 7.0% (95% CI 1.4-12.6) of the newborns had clinical signs of infection. In studies where mothers had PROM, 14.1% (95% CI 10.6-17.6) of the newborns had clinical signs of infection. In studies where mothers had PPROM, 32.1% (95% CI 3.7-60.6) of the newborns had clinical signs of infection. In studies where mothers had prolonged rupture of membranes, 18.6% (95% CI 0.4-36.7) of the newborns had clinical signs of infection (Figure 5). In studies that measured neonatal clinical signs or lab tests (“risk/lab&signs”), the prevalence of neonatal infection was even higher among newborns born to women with preterm labor (37.5%, 95% CI -3.1-78.2) or PROM (16.1%, 95% CI 1.3-31.0) although the 95% CIs overlapped with the studies that measured neonatal clinical signs only.

## Discussion

We estimate a high prevalence of neonatal infection in the first seven days of life where mothers had genital tract infection or risk factors for infection. In studies of pregnant mothers with laboratory confirmed infection, 17% of their newborns had positive laboratory cultures for infection. Similarly, in studies of pregnant mothers with clinical signs of infection, 20% of newborns had positive lab cultures for infection.

We included studies that measured maternal colonization or risk factors for infection. After excluding studies with known or possible antibiotic use, 1-7% (depending on definition of infection) of newborns exposed to maternal colonization developed neonatal infections. Most studies in our review that assessed maternal colonization examined GBS. Our findings are consistent with a US based review showing 2.0% of newborns exposed to maternal GBS developed early-onset GBS neonatal sepsis [128,129]. The lower prevalence of GBS colonization among newborns exposed to maternal GBS colonization in Europe compared to the US may be related to regional differences in GBS strains or a sampling bias in the studies meeting our inclusion criteria. Approximately 30-45% of newborns were surface colonized if they were born to a colonized mother, suggesting a high rate of surface bacterial transmission via direct contact through the birth canal between the mother and newborn. Pregnant women with risk factors, particularly PPROM, preterm labor, and prolonged rupture of membranes, had a high prevalence of neonatal infection. These risk factors may lead to bacterial infections, and may be indications for women to present to a health facility. In settings where laboratory or clinical measures are not available, the presence of risk factors may be a useful target for interventions to prevent early-onset neonatal sepsis.

Studies with lab tests for neonatal infection provided a more conservative measure of prevalence than studies with clinical signs of neonatal infection. The true prevalence of neonatal infection is likely to be between the specific lab-confirmed measures and sensitive clinical signs measures. Studies with colonization measures were also dependent on laboratory facilities and these findings may not be generalizable to populations without such facilities. Not many studies utilized molecular diagnostic methods in our review. As PCR-based diagnoses of maternal and neonatal infections are becoming more widely used, our ability to detect true neonatal infections will improve.

More studies are needed to accurately estimate the prevalence of early-onset neonatal infection in cases where mothers are infected or colonized, especially in low-income countries. Among our included studies, there was no maternal infection data (lab confirmed or clinical signs) from Africa and the eastern Mediterranean. In southeast Asia, no studies looked at lab-confirmed maternal infection and only two studies reported clinical signs of infection.

Our review has several limitations. Most studies were assessed to be at high risk or unclear risk of bias, which introduced systematic differences in baseline characteristics, outcome measurements, and estimates of point prevalence. We conducted a sensitivity analysis to exclude high risk studies in order to provide less biased estimates.

Since all studies were facilities-based, mostly concentrated in urban settings in the Americas and Europe, we could not capture the prevalence of early-onset neonatal sepsis among home births, rural births, or births at community facilities in lower-income countries, thereby limiting the generalizability of these findings. Included studies tended to have high prevalence of maternal infections, suggesting that these studies may be more biased towards pregnant women with more severe disease or risk factors. Authors are more likely to report positive findings in international English journals, whereas negative findings are published in non-English journals. We excluded non-English articles due to our limited resources. To assess for publication bias, we used funnel plots of standard error and prevalence to graph the correlation between the variance and distribution of effect sizes. Results were not statistically significant (p = 0.10).

There were limited data available on intrapartum antibiotic use in these studies. The majority of studies had either some or unknown antibiotic use. The inclusion of pregnant mothers who had received antibiotics would lead a study to underestimate the vertical transmission rates that would occur without antibiotic intervention.

There was significant heterogeneity of the prevalence results included in the meta-analysis because of the different measures of exposures and outcomes (lab-confirmed, clinical signs, colonization, and risk factors). To minimize heterogeneity, we grouped studies by exposure and outcome definitions and conducted separate meta-analyses for each group, although this reduced the number of studies in each meta-analysis. To account for additional differences, we used a random-effects model. We did not provide an overall estimate of vertical transmission across all studies because we assessed the studies to be too heterogeneous.

A strength of our study was our comprehensive search strategy; we included all articles with a measure or raw data on maternal and neonatal infections or colonization. Our study is the first to synthesize different measures of infection and provide prevalence estimates of neonatal early-onset infection among newborns of women with infections, colonization, or risk factors. Our findings highlight the need for better screening and diagnostics to identify pregnant women with infections and/or colonization to better understand the population based prevalence of maternal infections and/or colonization with common EOS pathogens in LMIC, which if treated would have the potential to reduce the burden of early-onset neonatal infections. Additional studies, such as a randomized controlled trial on the effect of intrapartum antibiotic prophylaxis on early-onset neonatal sepsis in low resource settings, are also needed.

## Conclusion

This study reinforces the importance of ongoing efforts in research and policy development to prevent early-onset neonatal sepsis by targeting pregnant women with infections (laboratory-confirmed, clinical signs), bacterial colonization, and risk factors. Standardizing definitions for maternal infections and newborns would be helpful to compare studies. High quality studies, with laboratory-confirmed, clinical signs, colonization, and risk factors are needed in low-resource areas, especially southeast Asia and Africa.

## Additional files

Additional file 1: Table S1. Search terms by database.
Additional file 2: Table S2. Studies included in systematic review and meta-analysis.
Additional file 3: Table S3. Studies included in systematic review and meta-analysis: Maternal exposure and neonatal outcome combinations.
Additional file 4: Table S4. Maternal exposure and neonatal outcome definitions and prevalences.
Additional file 5: Figure S1. Risk of bias summary.

## Abbreviations

CI: Confidence interval
GBS: Group B Streptococcus
IQR: Interquartile range
PROM: Premature rupture of membranes
PPROM: Preterm premature rupture of membranes
ROM: Rupture of membranes
WHO: World Health Organization
