# Supplementary material for: Prevalence of early-onset neonatal infection among newborns of mothers with bacterial infection or colonization: a systematic review and meta-analysis
Source: BMC Infect Dis. 2015 Mar 7;15:118. doi: 10.1186/s12879-015-0813-3 (PMC4364328; doi:10.1186/s12879-015-0813-3)
Supplement: Additional file 1: Table S1. — Search terms by database. [file 12879_2015_813_MOESM1_ESM.pdf]

Appendix

Table 1. Search terms by database

Keywords	Controlled Vocabulary	
	MeSH (Pubmed, Cochrane, Global Health)	Emtree
acute vaginitis; amniitis; amnionitides; amniotic infection; antenatal infection; antenatal infections; antepartum infection; antepartum infections; bacteremias; bacteremic shock; bacterial shock; bacterial vaginitides; bacterial vaginitis; bacterial vaginoses; bacterial vaginoses; bacterial vaginosis; bacterial vaginosis; bacteriemic shock; blood poisoning; blood poisonings; boils; bullous impetigo; BV; candida vaginitis; carbuncles; chlorioamnionitis; chorioamnionitides; clitoral abscesses; colibacillosis; coliform infection; coliform infections; colpitis; Corynebacterium vaginale; e coli; e coli infection; e coli infections; e. coli; e. coli infection; e. coli infections; endotoxemias; endotoxic shock; Escherichia infection; febrile disease; febrile reaction; febrile response; female genital tract infections; fevers; fungemias; funisitides; funisitis; furuncle; furuncles; furunculoses; GBS infection; GBS infections; genital tract infection; Genitourinary tract infection; group B beta haemolytic streptococcal infection; group B beta hemolytic streptococcal infection; group B beta hemolytic Streptococcus infection; group B streptococcal infections; group B streptococci infection; group b streptococcus; group B Streptococcus infection; gynecologic abscess; gynecologic abscesses; gynecological abscess; gynecological abscesses; gynecological infections; Haemophilus vaginalis; hemophilus vaginalis; hyperthermias; impetigo contagiosa; intrauterine infections; klebsiella infections; kolpitis; maternal bacteremia; maternal fever; maternal infection; maternal infections; maternal pyrexia; maternal sepsis; methicillin resistant staphylococcus aureus; methicillin resistant Staphylococcus aureus bacteraemia; methicillin resistant Staphylococcus aureus bacteremia; methicillin resistant Staphylococcus aureus pneumonia; methicillin resistant Staphylococcus aureus sepsis; methicillin resistant Staphylococcus aureus septicaemia; methicillin resistant Staphylococcus aureus septicemia; methicillin-resistant staphylococcus aureus; methicillin-resistant staphylococcus aureus infection; monilial vaginitides; monilial vaginitis; MRSA; MRSA bacteraemia; MRSA bacteremia; MRSA infection; MRSA infections; MRSA pneumonia; MRSA sepsis; MRSA septicaemia; MRSA septicemia; nonspecific vaginitis; obstetric infection; obstetric infections; obstetrical infection; obstetrical infections; ovary abscesses; postpartum infection; puerperal infections; pyaemia; Pyaemias; pyemia; pyemias; pyohemia; pyohemias; pyrexia; pyrexias; rectal carriage; rectal colonization; rectal colonization; s aureus; s. aureus; sepsis syndrome; sepsis syndromes; septic disease; septic shock; septic shock; septicemias; severe sepsis; SIRS; skin boil; skin boils; staph infection; staph infections; staphylococcal bacteraemia; staphylococcal dermatitis; staphylococcal infection; staphylococcal infections; staphylococcal sepsis; staphylococcal septicaemia; staphylococcal septicemia; staphylococcal skin disease; staphylococcal skin diseases; staphylococcoses; staphylococcosis; Staphylococcus aureus bacteraemia; Staphylococcus aureus bacteremia; Staphylococcus aureus infection; Staphylococcus aureus infections; Staphylococcus aureus sepsis; Staphylococcus aureus septicaemia; Staphylococcus aureus septicemia; Staphylococcus bacteraemia; Staphylococcus bacteremia; Staphylococcus dermatitis; staphylococcus infections; Staphylococcus sepsis; Staphylococcus septicaemia; Staphylococcus septicemia; Staphylococcus skin disease; Staphylococcus skin diseases; Staphylococcus skin infection; Staphylococcus skin infections; staphylodermatitis; strep infection; strep infections; streptococcal infection; streptococci infection; streptococci infections; streptococcoses; streptococcosis; streptococcus agalactiae infection; streptococcus agalactiae infections; streptococcus infections; toxic shock; toxic shock syndromes; tuboovarian abscesses; unknown origin fever; urinary infection; urinary tract carriage; urinary tract colonization; urinary tract colonization; urinary tract infection; urologic infection; uterine tube abscesses; UTI; UTIs; vagina infection; vagina inflammation; vaginal candidiasis; vaginal carriage; vaginal colonization; vaginal colonization; vaginal infection; vaginitides; vulva inflammation; vulval inflammation; vulvar inflammation; vulvovaginal candidiases; vulvovaginal candidiasis; vulvovaginal gland inflammation; vulvovaginal inflammation; vulvovaginal moniliases; vulvovaginal moniliasis; vulvovaginitides	Bacteremia*; Candidiasis, Vulvovaginal; Carbuncle; Chorioamnionitis; Endotoxemia; Escherichia coli Infections; Fever; Fever of Unknown Origin; Fungemia; Furunculosis; Gardnerella vaginalis; Impetigo; Klebsiella Infections; Methicillin-Resistant Staphylococcus aureus; Puerperal Infection; Pyrogens; Sepsis*; Shock, Septic; Staphylococcal Infections*; Staphylococcal Skin Infections*; Streptococcal Infections; Systemic Inflammatory Response Syndrome*; Urinary Tract Infections; Vaginitis*; Vaginosis, Bacterial; Vulvovaginitis	Amnionitis; Bacteremia*; candidemia; Candiduria; Carbuncle; Chorioamnionitis; clitoral abscess; endogenous pyrogen; Endotoxemia; Escherichia coli*; Escherichia coli infection*; female genital tract infection; Fever; Fungemia; Furunculosis; Gardnerella vaginalis; genital tract inflammation*; granuloma inguinale; group B streptococcal infection; group B streptococcal meningitis; group B streptococcal pneumonia; gynecological infection; hyperthermia; Impetigo; impetigo bullosa; impetigo herpetiformis; intrauterine infection *; klebsiella*; klebsiella infection*; klebsiella pneumoniae infection; meningococcemia; methicillin resistant staphylococcus aureus infection; ovary abscess; Puerperal Infection; pyrexia idiopathica; pyrogen*; Sepsis*; septicemia; Shock, Septic; staphylococcal bacteremia; staphylococcal scalded skin syndrome; staphylococcal skin infection; staphylococcus aureus*; staphylococcus infection*; streptococcus*; streptococcus infection*; Systemic Inflammatory Response Syndrome*; toxic shock syndrome; tuboovarian abscess; urine tract infection*; urosepsis; uterine tube abscess; vagina candidiasis; Vaginitis; Viremia; vulvitis; Vulvovaginitis
congenital transmission; fetomaternal infection transmission; infectious pregnancy complications; maternal fetal infection transmission; maternal fetal infectious disease transmission; maternal vertical transmission; maternal-fetal infection transmission; maternal-fetal infectious disease transmission; maternally acquired infection; mother fetus transmission; mother to child transmission; mother to infant transmission; mother-fetus	Infectious Disease Transmission, Vertical; Pregnancy Complications,	Intrauterine infection*; Vertical transmission

transmission; mother-to-child transmission; mother-to-infant transmission; vertical infection transmission; vertical infectious disease transmission; vertical pathogen transmission; vertically transmitted disease	Infectious*	
Bacillaemia; bacillemia; bacteraemia; Bacteremias; bacteremic shock; bacterial shock; bacteriemia; bacteriemic shock; blood poisoning; blood poisonings; candida fungaemia; candida fungemia; candida sepsis; candidaemia; early onset infection; early onset infections; early onset neonatal sepsis; early onset sepsis; early-onset infection; early-onset infections; early-onset neonatal sepsis; early-onset sepsis; Endotoxaemia; Endotoxemias; endotoxic shock; endotoxin shock; endotoxine shock; Endotoxinemia; endotoxinic shock; EONS; Fungaemia; fungal sepsis; Fungemia; Fungemias; meningococcaemia; meningococcal sepsis; meningococcal septic shock; meningococcal septicaemia; meningococcal septicemia; Neisseria meningitides bacteremia; Neisseria meningitides sepsis; neonatal bacteremia; neonatal infection; neonatal infections; neonatal meningitis; neonatal pneumonia; neonatal sepsis; neonatal septicemia; newborn infections; newborn meningitis; newborn pneumonia; newborn septicemia; Pyaemia; Pyaemias; pyemia; pyemias; pyohemia; pyohemias; sepsis syndrome; sepsis syndromes; Septicemias; septicemic shock; severe sepsis; SIRS; staphylococcal bacteraemia; staphylococcal sepsis; staphylococcal septicaemia; staphylococcal septicemia; Staphylococcus aureus bacteraemia; Staphylococcus aureus bacteremia; Staphylococcus aureus sepsis; Staphylococcus aureus septicaemia; Staphylococcus aureus septicemia; Staphylococcus bacteraemia; Staphylococcus bacteremia; Staphylococcus sepsis; Staphylococcus septicaemia; staphylococcus septicemia; toxic shock; toxic shock syndromes	Bacteremia*; Endotoxemia; Fungemia; Sepsis*; Shock, Septic; Systemic Inflammatory Response Syndrome*	Bacteremia*; Candidemia; Endotoxemia; Fungemia; Meningococcemia; Newborn infection; Newborn sepsis; Sepsis; Septic Shock; Septicemia; Staphylococcal bacteremia; Systemic Inflammatory Response Syndrome; Toxic shock syndrome

* Indicates controlled vocabulary searched as Mesh:NoExp in Pubmed/Cochrane/GH, and /de in Embase. All other controlled vocabulary searched as Mesh(Pubmed/Cochrane/GH) and /exp(Embase).
