# Supplementary material for: Prevalence of early-onset neonatal infection among newborns of mothers with bacterial infection or colonization: a systematic review and meta-analysis
Source: BMC Infect Dis. 2015 Mar 7;15:118. doi: 10.1186/s12879-015-0813-3 (PMC4364328; doi:10.1186/s12879-015-0813-3)
Supplement: Additional file 2: Table S2. — Studies included in systematic review and meta-analysis. [file 12879_2015_813_MOESM2_ESM.pdf]

Appendix Table 2. Studies included in systematic review and meta-analysis

Author	Year	Country	Study sample size	Study Type	Setting	Urban or rural	Timing of EOS diagnosis	Antibiotic use	Specialized population	WHO region	NMR	Income (USD)
AbeleHorn	1997	Germany	395	Cohort	Health facility	Urban	Unknown	Not used	Other	EUR	2	43110
Adriaanse	1995	Netherlands	1020	Cohort	Multi-center	Urban	≤7 days	Not used	All	EUR	3	49050
Andrews	2008	USA	5732	Cohort	Health facility	Urban	≤7 days	Unknown	All	AMR	4	47390
Averbuch	1995	Israel	90	Cohort	Health facility	Urban	Unknown	Unknown	PPROM	EUR	2	27170
Ayata	1994	Turkey	114	Cohort	Health facility	Urban	≤7 days	Unknown	All	EUR	12	9890
Ayengar	1991	India	1792	Cohort	Health facility	Urban	≤7 days	Unknown	All	SEAR	34	1330
Berardi	2011	Italy	4699	Cohort	Multi-center	Urban	Unknown	Used	All	EUR	2	35150
Blott	1988	UK	30	Cohort	Health facility	Urban	≤7 days	Unknown	Prolonged ROM	EUR	3	38370
Bobitt	1985	USA	937	Cohort	Health facility	Urban	Unknown	Used	All	AMR	4	47390
Bobitt	1977	USA	12	Cohort	Health facility	Urban	Unknown	Unknown	PT/PROM	AMR	4	47390
Bourgeois-Nicolaos	2010	France	1139	Cohort	Health facility	Urban	≤7 days	Unknown	All	EUR	2	42390
Boyer	1981	USA	924	Cohort	Health facility	Urban	≤7 days	Not used	PT/PROM	AMR	4	47390
Broekhuizen	1985	USA	53	Cohort	Health facility	Urban	Unknown	Unknown	PPROM	AMR	4	47390
Buckler	2010	USA	242	Cohort	Health facility	Urban	Unknown	Used	All	AMR	4	47390
Burman	1992	Sweden	4559	Cohort	Multi-center	Unknown	Unknown	Not used	All	EUR	2	50110
Canpolat	2011	Turkey	74	Cohort	Health facility	Urban	≤7 days	Used	PPROM	EUR	12	9890
Cararach	1998	Spain	733	Cohort	Multi-center	Unknown	≤7 days	Unknown	PROM	EUR	2	31750
Carlan*	1997	USA	93	Cohort	Health facility	Urban	≤7 days	Used	PPROM	AMR	4	47390
Christensen	1982	Sweden	300	Cohort	Health facility	Unknown	≤7 days	Unknown	All	EUR	2	50110
Christmas	1992	USA	94	Cohort	Health facility	Unknown	Unknown	Used	PPROM	AMR	4	47390
Coultrip	1994	USA	89	Cohort	Multi-center	Urban	Unknown	Used	Preterm	AMR	4	47390
Craig	1996	Australia	24	Cohort	Health facility	Urban	Unknown	Used	All	WPR	3	
Cutland	2009	South Afric	8011	Cohort	Health facility	Urban	≤7 days	Used	All	AFR	19	6090
de Araujo	1994	Brazil	223	Cohort	Health facility	Urban	≤7 days	Used	All	AMR	12	9390
Dollner	2002	Norway	221	Cohort	Health facility	Urban	≤7 days	Used	All	EUR	2	84290
Dudley	1991	Australia	81	Cohort	Health facility	Urban	≤7 days	Used	PPROM	WPR	3	
Dutta	2010	India	728	Cohort	Health facility	Urban	≤7 days	Used	Preterm	SEAR	34	1330
Easmon	1985	UK	1457	Cohort	Health facility	Urban	Unknown	Used	All	EUR	3	38370
Elder*	1971	USA	9156	Cohort	Health facility	Urban	Unknown	Not used	All	AMR	4	47390
El-Kersh	2002	Saudi Arabi	217	Cohort	Health facility	Urban	≤7 days	Not used	All	EMR	12	
Elzbieta	2009	Poland	100	Cohort	Health facility	Unknown	≤7 days	Used	All	EUR	4	12440
Eren	2005	Turkey	500	Cohort	Health facility	Urban	≤7 days	Unknown	Other	EUR	12	9890
Faro	2010	USA	2108	Cohort	Multi-center	Urban	≤7 days	Used	All	AMR	4	47390
Feinstein	1986	USA	146	Cohort	Health facility	Urban	Unknown	Not used	PROM	AMR	4	47390
Franciosi	1973	USA	1020	Cohort	Health facility	Unknown	≤7 days	Unknown	All	AMR	4	47390
Frederiksen*	1992	Denmark	30	Cohort	Community	Unknown	≤7 days	Unknown	All	EUR	2	59050
Gauthier	1994	USA	225	Cohort	Health facility	Urban	Unknown	Not used	PPROM	AMR	4	47390
Gerard	1979	Belgium	1115	Cohort	Health facility	Urban	≤7 days	Unknown	All	EUR	2	45910
Ghanim	2011	USA	6626	Cohort	Health facility	Urban	Unknown	Used	All	AMR	4	47390

Appendix Table 2. Studies included in systematic review and meta-analysis (continued)

Author	Year	Country	Study sample size	Study Type	Setting	Urban or rural	Timing of EOS diagnosis	Antibiotic use	Specialized population	WHO region	NMR	Income (USD)
Gibbs	1981	USA	48	Cohort	Health facility	Unknown	Unknown	Used	All	AMR	4	47390
Gibbs	1988	USA	48	Cohort	Health facility	Unknown	≤7 days	Used	Other	AMR	4	47390
Gilbert	2005	UK centers	8797	Cohort	Multi-center	Unknown	Unknown	Not used	PT/PROM	EUR	3	38370
Goldenberg	2008	USA	351	Cohort	Health facility	Unknown	≤7 days	Unknown	Preterm	AMR	4	47390
Graham	1982	USA	9069	Cohort	Health facility	Urban	Unknown	Used	PPROM	AMR	4	47390
Hashavya	2011	Israel	1648	Cohort	Health facility	Urban	≤7 days	Used	All	EUR	2	27170
Hervas	1993	Spain	1003	Cohort	Health facility	Urban	Unknown	Unknown	All	EUR	2	31750
Hickman	1999	USA	546	Cohort	Multi-center	Urban	≤7 days	Used	All	AMR	4	47390
Hyckel	1992	Germany	18	Cohort	Health facility	Urban	≤7 days	Not used	PPROM	EUR	2	43110
Itakura	1996	Japan	1280	Cohort	Health facility	Urban	≤7 days	Unknown	All	WPR	1	41850
Kadanali	2005	Turkey	150	Cohort	Health facility	Urban	≤7 days	Not used	All	EUR	12	9890
Kafetzis	2004	Greece	251	Cohort	Health facility	Urban	≤7 days	Unknown	All	EUR	2	26940
Kalinka	2006	Poland	120	Cohort	Health facility	Urban	≤7 days	Not used	All	EUR	4	12440
Kappy	1979	USA	188	Cohort	Health facility	Urban	Unknown	Used	PROM	AMR	4	47390
Kasper	2010	Austria	118	Cohort	Health facility	Urban	≤7 days	Unknown	Preterm	EUR	2	47060
Kishore	1987	India	109	Cohort	Health facility	Urban	≤7 days	Not used	All	SEAR	34	1330
Koh	1979	USA	26129	Cohort	Health facility	Urban	Unknown	Used	All	AMR	4	47390
Kollee	1989	Netherlands	632	Cohort	Health facility	Urban	≤7 days	Unknown	All	EUR	3	49050
Kordek	2006	Poland	46	Cohort	Health facility	Urban	≤7 days	Used	PT/PROM	EUR	4	12440
Kordek	2011	Poland	286	Cohort	Health facility	Urban	≤7 days	Used	All	EUR	4	12440
Kunze*	2006	Germany	1438	Cohort	Health facility	Urban	≤7 days	Used	All	EUR	2	43110
Kunze	2011	Germany	869	Cohort	Health facility	Urban	≤7 days	Used	All	EUR	2	43110
Liang	1986	Hong Kong	168	Cohort	Health facility	Urban	≤7 days	Not used	All	WPR	1	
Lijoi	2007	Italy	2158	Cohort	Health facility	Urban	≤7 days	Not used	All	EUR	2	35150
Lim	1997	Malaysia	196	Cohort	Health facility	Urban	≤7 days	Unknown	All	WPR	3	7760
Matorras	1991	Spain	1050	Cohort	Health facility	Urban	≤7 days	Not used	All	EUR	2	31750
Matsubara	2002	Japan	583	Cohort	Health facility	Urban	Unknown	Unknown	All	WPR	1	41850
Matsuda	1995	Japan	41	Cohort	Health facility	Urban	Unknown	Unknown	Preterm	WPR	1	41850
McCaul	1992	USA	367	Cohort	Health facility	Urban	Unknown	Not used	PT/PROM	AMR	4	47390
McGrady*	1985	USA	1342	Cohort	Community	Mixed	Unknown	Unknown	All	AMR	4	47390
McLauchlin*	1990	UK	248	Population	Community	Mixed	≤7 days	Unknown	All	EUR	3	38370
Mercer	1999	USA	8474	Cohort	Multi-center	Urban	≤7 days	Used	All	AMR	4	47390
Mercer	1997	USA	1867	Cohort	Multi-center	Unknown	≤7 days	Not used	PPROM	AMR	4	47390
Merenstein	1980	USA	1815	Cohort	Unknown	Unknown	≤7 days	Not used	All	AMR	4	47390
Mitra	1997	India	100	Cohort	Unknown	Unknown	≤7 days	Used	PROM	SEAR	34	1330
Mitsuda	1996	Japan	466	Cohort	Health facility	Urban	≤7 days	Unknown	All	WPR	1	41850
Morales*	1989	USA	212	Cohort	Multi-center	Urban	Unknown	Not used	PPROM	AMR	4	47390
Morales	1987	USA	260	Cohort	Health facility	Urban	≤7 days	Used	PPROM	AMR	4	47390
Morales	1986	USA	1207	Cohort	Unknown	Unknown	Unknown	Not used	All	AMR	4	47390

Appendix Table 2. Studies included in systematic review and meta-analysis (continued)

Author	Year	Country	Study sample size	Study Type	Setting	Urban or rural	Timing of EOS diagnosis	Antibiotic use	Specialized population	WHO region	NMR	Income (USD)
Muthusami	2007	India	77	Cohort	Health facility	Urban	≤7 days	Unknown	All	SEAR	34	1330
Nadisauskiene	1996	Lithuania	102	Cohort	Health facility	Urban	Unknown	Not used	Preterm	EUR	3	11390
Namavar Jahromi	2008	Iran	1197	Cohort	Health facility	Urban	≤7 days	Used	All	EMR	19	2500
Natale	1995	Italy	7176	Cohort	Multi-center	Unknown	≤7 days	Used	All	EUR	2	35150
Newton	1989	USA	2908	Cohort	Health facility	Urban	Unknown	Used	All	AMR	4	47390
Niduvaje	2006	Singapore	4553	Cohort	Health facility	Urban	≤7 days	Used	All	WPR	1	40070
Nolla-Salas*	1998	Spain	15	Population	Multi-center	Urban	≤7 days	Unknown	All	EUR	2	31750
Orrett	2003	Trinidad	201	Cohort	Multi-center	Unknown	≤7 days	Unknown	All	AMR	23	15380
Papantoniou	1997	Greece	32	Cohort	Health facility	Urban	Unknown	Used	PPROM	EUR	2	26940
Pass	1982	USA	68	Cohort	Multi-center	Urban	Unknown	Used	All	AMR	4	47390
Pearson*	1967	USA	12000	Population	Health facility	Urban	Unknown	Unknown	All	AMR	4	47390
Persson*	1986	Sweden	1786	Nested	Health facility	Urban	≤7 days	Used	All	EUR	2	50110
Philip	1982	USA	524	Cohort	Health facility	Urban	≤7 days	Unknown	All	AMR	4	47390
Pinter	2009	USA	317	Cohort	Health facility	Urban	≤7 days	Unknown	All	AMR	4	47390
Piper	1999	USA	1046	Cohort	Health facility	Urban	Unknown	Used	All	AMR	4	47390
Puchner	1993	Austria	80	Cohort	Unknown	Unknown	Unknown	Not used	All	EUR	2	47060
Puopolo	2011	USA	1413	Nested	Multi-center	Unknown	≤7 days	Used	All	AMR	4	47390
Pylipow	1994	USA	2040	Cohort	Health facility	Urban	≤7 days	Used	All	AMR	4	47390
Quentin*	1989	France	114	Cohort	Health facility	Urban	Unknown	Unknown	PPROM	EUR	2	42390
Regan	1996	USA	13646	Cohort	Multi-center	Urban	Unknown	Not used	All	AMR	4	47390
Reid	1975	UK	369	Cohort	Health facility	Urban	Unknown	Used	All	EUR	3	38370
Rosemond	1995	USA	224	Cohort	Health facility	Urban	Unknown	Used	PPROM	AMR	4	47390
Saez-Llorens	1995	Panama	800	Cohort	Health facility	Urban	≤7 days	Not used	PT/PROM	AMR	10	6970
Sensini	1997	Italy	2300	Cohort	Health facility	Urban	≤7 days	Unknown	All	EUR	2	35150
Seoud	2010	Lebanon	779	Cohort	Multi-center	Urban	Unknown	Used	All	EMR	7	8880
Simor	1990	Canada	10	Cohort	Health facility	Urban	≤7 days	Unknown	All	AMR	4	43270
Smith*	2009	Denmark	37	Cohort	Health facility	Urban	Unknown	Used	All	EUR	2	59050
Sperling	1987	USA	264	Cohort	Health facility	Urban	≤7 days	Used	Other	AMR	4	47390
Spinnato	1987	USA	114	Cohort	Health facility	Urban	Unknown	Unknown	PPROM	AMR	4	47390
Suara	1994	Gambia	196	Cohort	Health facility	Unknown	≤7 days	Unknown	All	AFR	32	7740
Syrogiannopoulos	1990	Greece	640	Cohort	Health facility	Unknown	≤7 days	Unknown	All	EUR	2	26940
Tafari	1979	Ethiopia	1351	Cohort	Health facility	Urban	≤7 days	Unknown	All	AFR	36	390
Towers	1990	USA	131	Cohort	Health facility	Urban	Unknown	Used	PT/PROM	AMR	4	47390
Tsolia	2003	Greece	1014	Cohort	Multi-center	Urban	≤7 days	Used	All	EUR	2	26940
Tuppurainen*	1989	Finland	8977	Cohort	Health facility	Urban	≤7 days	Not used	All	EUR	2	47720
Varner	1981	USA	116	Cohort	Health facility	Urban	Unknown	Not used	PROM	AMR	4	47390
Vergani*	2002	USA	32630	Cohort	Health facility	Urban	≤7 days	Not used	All	AMR	4	47390
Visconti	1985	Italy	1516	Cohort	Multi-center	Urban	Unknown	Unknown	All	EUR	2	35150
Volumenie	2001	France	5374	Cohort	Health facility	Urban	Unknown	Used	All	EUR	2	42390

Appendix Table 2. Studies included in systematic review and meta-analysis (continued)

Author	Year	Country	Study	Study	Setting	Urban or	Timing of	Antibiotic	Specialized	WHO	NMR	Income
Wallace	1983	USA	11	Cohort	Health facility	Urban	≤7 days	Used	Preterm	AMR	4	47390
Weintraub	1983	Israel	385	Cohort	Health facility	Urban	≤7 days	Unknown	All	EUR	2	27170
Wilson	1982	USA	143	Cohort	Health facility	Urban	≤7 days	Used	PPROM	AMR	4	47390
Wood*	1981	USA	569	Cohort	Multi-center	Urban	Unknown	Unknown	All	AMR	4	47390
Yoon	2000	South Korea	315	Cohort	Health facility	Urban	≤7 days	Unknown	Preterm	WPR	2	17890

* 15 studies were not included in the quantitative meta-analyses, but were included in the qualitative assessment.

NMR:Neonatal mortality rate (per 1000 live births); EOS: early-onset sepsis; LBW: low birth weight; PROM: premature rupture of membrane;

PPROM: preterm premature rupture of membrane; PT/PROM: Preterm or premature rupture of membrane

WHO: World Health Organization; AFR: Africa; AMR: Americas; EMR: East Mediterrean; EUR: Europe; SEAR: South East Asia; WPR: Western Pacific
