# Supplementary material for: Prevalence of early-onset neonatal infection among newborns of mothers with bacterial infection or colonization: a systematic review and meta-analysis
Source: BMC Infect Dis. 2015 Mar 7;15:118. doi: 10.1186/s12879-015-0813-3 (PMC4364328; doi:10.1186/s12879-015-0813-3)
Supplement: Additional file 3: Table S3. — Studies included in systematic review and meta-analysis: Maternal exposure and neonatal outcome combinations. [file 12879_2015_813_MOESM3_ESM.pdf]

**Appendix Table 3. Studies included in systematic review and meta-analysis: Maternal exposure and neonatal outcome combinations**

| Author | Year | Maternal infection and Neonatal infection | Maternal colonization and neonatal infection | Maternal colonization and neonatal colonization | Maternal risk factors and neonatal infection | Other |
|---|---|---|---|---|---|---|
| AbeleHorn | 1997 | | Meta-analysis | Meta-analysis | | |
| Adriaanse | 1995 | | | Meta-analysis | | |
| Andrews | 2008 | | Meta-analysis | | | |
| Averbuch | 1995 | Meta-analysis | | | | |
| Ayata | 1994 | | | Meta-analysis | | |
| Ayengar | 1991 | | | Meta-analysis | | |
| Berardi | 2011 | | | Meta-analysis | | |
| Blott | 1988 | | | | Meta-analysis | |
| Bobitt | 1985 | | Meta-analysis | | | |
| Bobitt | 1977 | Meta-analysis | | | Meta-analysis | |
| Bourgeois- | 2010 | | | Meta-analysis | | |
| Boyer | 1981 | | Meta-analysis | | | |
| Broekhuizen | 1985 | Meta-analysis | | | | |
| Buckler | 2010 | | Meta-analysis | | | |
| Burman | 1992 | | Meta-analysis | | | |
| Canpolat | 2011 | | | | Meta-analysis | |
| Cararach | 1998 | | | | Meta-analysis | |
| Carlan* | 1997 | | | | | Qualitative |
| Christensen | 1982 | | Meta-analysis | | | |
| Christmas | 1992 | | | | Meta-analysis | |
| Coultrip | 1994 | Meta-analysis | | | Meta-analysis | |
| Craig | 1996 | Meta-analysis | Descriptive | Descriptive | | |
| Cutland | 2009 | | | Meta-analysis | | |
| de Araujo | 1994 | Meta-analysis | | | | |
| Dollner | 2002 | Meta-analysis | | | | |
| Dudley | 1991 | Meta-analysis | | | | |
| Dutta | 2010 | Meta-analysis | | | Meta-analysis | |
| Easmon | 1985 | | | Meta-analysis | | |
| Elder* | 1971 | Qualitative | | | | |
| El-Kersh | 2002 | | | Meta-analysis | | |
| Elzbieta | 2009 | | | Meta-analysis | | |
| Eren | 2005 | | | Meta-analysis | | |
| Faro | 2010 | | Meta-analysis | | | |
| Feinstein | 1986 | Meta-analysis | | | Meta-analysis | |
| Franciosi | 1973 | | | Meta-analysis | | |
| Frederiksen* | 1992 | | | | | Qualitative |
| Gauthier | 1994 | Meta-analysis | | | Meta-analysis | |
| Gerard | 1979 | | | Meta-analysis | | |

**Appendix Table 3. Studies included in systematic review and meta-analysis: Maternal exposure and neonatal outcome combinations (continued)**

| Author | Year | Maternal infection and Neonatal infection | Maternal colonization and neonatal infection | Maternal colonization and neonatal colonization | Maternal risk factors and neonatal infection | Other |
|---|---|---|---|---|---|---|
| Ghanim | 2011 | | Meta-analysis | | | |
| Gibbs | 1981 | Meta-analysis | | | | |
| Gibbs | 1988 | Meta-analysis | | | | |
| Gilbert | 2005 | | | | Meta-analysis | |
| Goldenberg | 2008 | Meta-analysis | | | | |
| Graham | 1982 | | | | Meta-analysis | |
| Hashavya | 2011 | | Meta-analysis | | | |
| Hervas | 1993 | | | Meta-analysis | | |
| Hickman | 1999 | | | Meta-analysis | | |
| Hvckel | 1992 | | | | Meta-analysis | |
| Itakura | 1996 | | Meta-analysis | | Meta-analysis | |
| Kadanali | 2005 | | | Meta-analysis | | |
| Kafetzis | 2004 | | | Meta-analysis | | |
| Kalinka | 2006 | | Meta-analysis | | | |
| Kappy | 1979 | | | | Meta-analysis | |
| Kasper | 2010 | Meta-analysis | | | | |
| Kishore | 1987 | | Meta-analysis | Meta-analysis | | |
| Koh | 1979 | Meta-analysis | | | | |
| Kollee | 1989 | | | Meta-analysis | | |
| Kordek | 2006 | Meta-analysis | | | Meta-analysis | |
| Kordek | 2011 | Meta-analysis | | | Meta-analysis | |
| Kunze* | 2006 | | | | | Qualitative |
| Kunze | 2011 | | | Meta-analysis | | |
| Liang | 1986 | | Meta-analysis | Meta-analysis | | |
| Lijoi | 2007 | | | Meta-analysis | | |
| Lim | 1997 | | Meta-analysis | | | |
| Matorras | 1991 | | Meta-analysis | Meta-analysis | | |
| Matsubara | 2002 | | Meta-analysis | | | |
| Matsuda | 1995 | Meta-analysis | | | | |
| McCaul | 1992 | | | | Meta-analysis | |
| McGrady* | 1985 | Qualitative | | | | |
| McLauchlin* | 1990 | | | | | Qualitative |
| Mercer | 1999 | Meta-analysis | | | Meta-analysis | |
| Mercer | 1997 | | Meta-analysis | | Meta-analysis | |
| Merenstein | 1980 | | | Meta-analysis | | |
| Mitra | 1997 | Meta-analysis | Meta-analysis | | | |
| Mitsuda | 1996 | | | Meta-analysis | | |
| Morales* | 1989 | | | | | Qualitative |
| Morales | 1987 | | Meta-analysis | | | |

**Appendix Table 3. Studies included in systematic review and meta-analysis: Maternal exposure and neonatal outcome combinations (continued)**

| Author | Year | Maternal infection and Neonatal infection | Maternal colonization and neonatal infection | Maternal colonization and neonatal colonization | Maternal risk factors and neonatal infection | Other |
|---|---|---|---|---|---|---|
| Morales | 1986 | | Meta-analysis | | | |
| Muthusami | 2007 | | Meta-analysis | | | |
| Nadisauskiene | 1996 | | | | Meta-analysis | |
| Namavar Jahromi | 2008 | | Meta-analysis | Meta-analysis | | |
| Natale | 1995 | | Meta-analysis | Meta-analysis | | |
| Newton | 1989 | Meta-analysis | | | | |
| Niduvaje | 2006 | | Meta-analysis | | | |
| Nolla-Salas* | 1998 | Qualitative | | | | |
| Orrett | 2003 | | Meta-analysis | | | |
| Papantoniou | 1997 | Meta-analysis | | | | |
| Pass | 1982 | Meta-analysis | | | | |
| Pearson* | 1967 | Qualitative | | | | |
| Persson* | 1986 | | | | | Qualitative |
| Philip | 1982 | Meta-analysis | | | Meta-analysis | |
| Pinter | 2009 | | | Meta-analysis | | |
| Piper | 1999 | | Meta-analysis | | | |
| Puchner | 1993 | Meta-analysis | | | | |
| Puopolo | 2011 | Meta-analysis | Meta-analysis | | Meta-analysis | |
| Pylipow | 1994 | | Meta-analysis | Meta-analysis | | |
| Quentin* | 1989 | | | | | Qualitative |
| Regan | 1996 | | Meta-analysis | | | |
| Reid | 1975 | | Meta-analysis | Meta-analysis | | |
| Rosemond | 1995 | Meta-analysis | | | | |
| Saez-Llorens | 1995 | | | Meta-analysis | Meta-analysis | |
| Sensini | 1997 | | Meta-analysis | Meta-analysis | | |
| Seoud | 2010 | | | Meta-analysis | | |
| Simor | 1990 | | Meta-analysis | | | |
| Smith* | 2009 | | | | | Qualitative |
| Sperling | 1987 | Meta-analysis | | | | |
| Spinnato | 1987 | | | | Meta-analysis | |
| Suara | 1994 | | | Meta-analysis | | |
| Syrogiannopoul | 1990 | | Meta-analysis | Meta-analysis | | |
| Tafari | 1979 | | | | Meta-analysis | |
| Towers | 1990 | | Meta-analysis | | | |
| Tsolia | 2003 | | | Meta-analysis | | |
| Tuppurainen* | 1989 | | | | | Qualitative |
| Varner | 1981 | | | | Meta-analysis | |

**Appendix Table 3. Studies included in systematic review and meta-analysis: Maternal exposure and neonatal outcome combinations (continued)**

| Author | Year | Maternal infection and Neonatal infection | Maternal colonization and neonatal infection | Maternal colonization and neonatal colonization | Maternal risk factors and neonatal infection | Other |
|---|---|---|---|---|---|---|
| Vergani* | 2002 | | | | | Qualitative |
| Visconti | 1985 | | | Meta-analysis | | |
| Volumenie | 2001 | | Meta-analysis | | | |
| Wallace | 1983 | Meta-analysis | | | | |
| Weintraub | 1983 | | | Meta-analysis | | |
| Wilson | 1982 | Meta-analysis | | | Meta-analysis | |
| Wood* | 1981 | Qualitative | | | | |
| Yoon | 2000 | Meta-analysis | | | | |

* 15 studies were not included in the quantitative meta-analyses, but were included in the qualitative assessment.
