# Supplementary material for: Prevalence of early-onset neonatal infection among newborns of mothers with bacterial infection or colonization: a systematic review and meta-analysis
Source: BMC Infect Dis. 2015 Mar 7;15:118. doi: 10.1186/s12879-015-0813-3 (PMC4364328; doi:10.1186/s12879-015-0813-3)
Supplement: Additional file 4: Table S4. — Maternal exposure and neonatal outcome definitions and prevalences. [file 12879_2015_813_MOESM4_ESM.pdf]

Appendix Table 4. Maternal exposure and neonatal outcome definitions and prevalences

Author	Year	Maternal lab confirmed infection		Maternal clinical signs		Maternal colonization		Neonatal lab confirmed sepsis		Neonatal clinical signs of sepsis		Neonatal colonization		Neonatal infection or clincal signs	
		**Definition**	**Prev**	**Definition**	**Prev**	**Definition**	**Prev**	**Definition**	**Prev**	**Definition**	**Prev**	**Definition**	**Prev**	**Definition**	**Prev**
AbeleHorn	1997					U. urealyticum vagina culture	0.67								
Adriaanse	1995					GBS anorectum, introital, or cervical culture	0.19					GBS ear, pharynx, umbilicus culture	0.11		
Andrews	2008					MRSA anus, vagina culture	0.15	MRSA culture	0.00						
Averbuch	1995	amniotic fluid culture or gram stain	0.67					blood culture	0.12						
Ayata	1994					GBS rectum, vagina introitus and cervical swabs	0.09					GBS throat, ear, umbilicus, conjuctival sac, skin culture	0.04		
Ayengar	1991					high vagina swabs	0.57								
Berardi	2011					GBS swab culture	0.25					GBS ear, pharynx, rectum culture	0.14		
Blott	1988							undefined in paper	0.07						
Bobitt	1985					GBS swab culture	0.11			clinical signs	0.04				
Bobitt	1977	blood and amniotic fluid cultures	0.58					blood of csf culture	0.17						

Appendix Table 4. Maternal exposure and neonatal outcome definitions and prevalences (continued)

Author	Year	Maternal lab confirmed infection		Maternal clinical signs		Maternal colonization		Neonatal lab confirmed sepsis		Neonatal clinical signs of sepsis		Neonatal colonization		Neonatal infection or clincal signs	
		Definition	Prev	Definition	Prev	Definition	Prev	Definition	Prev	Definition	Prev	Definition	Prev	Definition	Prev
Bourgeois-Nicolaos	2010					S. aureus vagina culture	0.06					S. aureus ear, gastric fluid culture	0.04		
Boyer	1981					GBS vagina culture	0.17	undefined in paper	0.00						
Broekhuizen	1985	amniotic fluid culture or gram stain, placental	0.28					blood culture	0.04						
Buckler	2010							blood culture	0.00						
Burman	1992					GBS vagina culture	0.18	GBS blood or csf culture	0.00						
Canpolat	2011									clinical signs	0.43				
Cararach	1998														
Carlan	1997														
Christensen	1982													blood culture and pneumonia	0.06
Christmas	1992									clinical and radiographi c findings of pneumonia	0.09				
Coultrip	1994	amniotic fluid culture	0.13												
Craig	1996					placenta, vagina swab	0.00					listeria gastric aspirate and surface culture	0.68		

Appendix Table 4. Maternal exposure and neonatal outcome definitions and prevalences (continued)

Author	Year	Maternal lab confirmed infection		Maternal clinical signs		Maternal colonization		Neonatal lab confirmed sepsis		Neonatal clinical signs of sepsis		Neonatal colonization		Neonatal infection or clincal signs	
		Definition	Prev	Definition	Prev	Definition	Prev	Definition	Prev	Definition	Prev	Definition	Prev	Definition	Prev
Cutland	2009					GBS vagina culture	0.21								
de Araujo	1994		0.27												
Dollner	2002			chorio	0.29					clinical signs	0.05				
Elder	1971	urine culture	0.05												
El-Kersh	2002					GBS high vagina, low vagina, rectum swabs	0.28					GBS throat, nose, ear, eye, rectal, umbilical culture	0.21		
Elzbieta	2009					GBS vagina swab	0.19					GBS nose culture	0.04		
Eren	2005					GBS vagina and rectum swab	0.09					GBS throat, umbilical culture	0.02		
Faro	2010					GBS lower vagina, perineum, external anal sphincter swab	0.29	GBS blood culture	0.00						
Feinstein	1986	amniotic fluid culture	0.24												
Franciosi	1973					vagina, anal, throat swab	0.05					GBS throat culture	0.01		
Frederiksen	1992														
Gauthier	1994	U. urealyticum only positive amniotic fluid	0.15					blood, urine, csf culture	0.01						
Gerard	1979					GBS vagina culture	0.07								

Appendix Table 4. Maternal exposure and neonatal outcome definitions and prevalences (continued)																
**Author**	**Year**	**Maternal lab confirmed infection**		**Maternal clinical signs**		**Maternal colonization**		**Neonatal lab confirmed sepsis**		**Neonatal clinical signs of sepsis**		**Neonatal colonization**		**Neonatal infection or clincal signs**	
		**Definition**	**Prev**	**Definition**	**Prev**	**Definition**	**Prev**	**Definition**	**Prev**	**Definition**	**Prev**	**Definition**	**Prev**	**Definition**	**Prev**
Ghanim	2011					GBS and S. aureus rectovagina culture	0.00								
Gibbs	1981														
Gibbs	1988							blood, csf culture	0.09						
Gilbert	2005							blood culture	0.05						
Goldenberg	2008							umbilical cord blood culture	0.23						
Graham	1982														
Hashavya	2011							blood, csf culture	0.00						
Hervas	1993					GBS vagina culture	0.07								
Hickman	1999					GBS vagina and rectum swab	0.28					GBS throat, rectum, umbilicus culture	0.14		
Hvckel	1992							blood culture	0.10						
Itakura	1996														
Kadanali	2005					GBS vagina and rectum culture	0.32					GBS ear, throat, umbilicus culture	0.17		

Appendix Table 4. Maternal exposure and neonatal outcome definitions and prevalences (continued)

Author	Year	Maternal lab confirmed infection		Maternal clinical signs		Maternal colonization		Neonatal lab confirmed sepsis		Neonatal clinical signs of sepsis		Neonatal colonization		Neonatal infection or clincal signs	
		Definition	Prev	Definition	Prev	Definition	Prev	Definition	Prev	Definition	Prev	Definition	Prev	Definition	Prev
Kafetzis	2004					vagina secretion	0.37					U. urealyticum rhinopharyngeal secretion or tracheal lavage culture	0.17		
Kalinka	2006					vagina colonization with m. hominis, u. urealyticum, bacterial vaginosis	0.43							blood, urine culture or clinical signs	0.18
Kappy	1979											surface culture; gastric aspirate gram stain	0.26		
Kasper	2010	amniotic fluid culture PCR	0.27							inflammatory markers and clinical signs	0.21				
Kishore	1987					vagina culture	0.70					ear, nose, skin culture	0.44		
Koh	1979			chorio	0.01										
Kollee	1989					GBS cervical and anal swab	0.18					nose, ear, anus, umbilicus culture	0.15		

Appendix Table 4. Maternal exposure and neonatal outcome definitions and prevalences (continued)

Author	Year	Maternal lab confirmed infection		Maternal clinical signs		Maternal colonization		Neonatal lab confirmed sepsis		Neonatal clinical signs of sepsis		Neonatal colonization		Neonatal infection or clincal signs	
		Definition	Prev	Definition	Prev	Definition	Prev	Definition	Prev	Definition	Prev	Definition	Prev	Definition	Prev
Kordek	2006													blood culture or clinical signs	0.33
Kordek	2011														
Kunze	2006														
Kunze	2011					GBS rectovagina culture	0.21					GBS external canal culture	0.02		
Liang	1986					GBS endocervix, perineum and rectum cultures	0.19					GBS ear, nose, throat, umbilicus culture	0.20		
Lijoi	2007					GBS vagina, rectum or urine culture	0.16					GBS ear, nose, pharynx culture	0.03		
Lim	1997					GBS vagina and rectum culture	0.10								
Matorras	1991					GBS vagina and rectum culture	0.12			undefined in paper	0.14				
Matsubara	2002					GBS vagina culture	0.08	undefined in paper	0.00						
Matsuda	1995		0.20												
McCaul	1992														
McGrady	1985	urinary tract infection	0.00												
McLauchlin	1990														
Mercer	1999							blood, csf culture	0.01						

Appendix Table 4. Maternal exposure and neonatal outcome definitions and prevalences (continued)

Author	Year	Maternal lab confirmed infection		Maternal clinical signs		Maternal colonization		Neonatal lab confirmed sepsis		Neonatal clinical signs of sepsis		Neonatal colonization		Neonatal infection or clincal signs	
		Definition	Prev	Definition	Prev	Definition	Prev	Definition	Prev	Definition	Prev	Definition	Prev	Definition	Prev
Mercer	1997					GBS vagina culture	0.19							blood culture or clinical signs	0.06
Merenstein	1980					GBS vagina and cervical culture	0.12					GBS ear, throat culture	0.04		
Mitra	1997							blood culture	0.19						
Mitsuda	1996					S. aureus vagina culture	0.08					S. aureus nose culture	0.10		
Morales	1989					GBS cervical culture	0.29								
Morales	1987					GBS vagina culture	0.32								
Morales	1986					GBS vagina culture	0.22								
Muthusami	2007					vagina swab	0.32			clinical signs	0.29				
Nadisauskiene	1996							undefined in paper	0.66						
Namavar Jahromi	2008					GBS rectovagina culture	0.09					skin, ear, mouth, throat culture	0.06		
Natale	1995					GBS vagina and rectum culture	0.11					GBS ear, phrynx culture	0.04		
Newton	1989			intra-amniotic infection based on clinical signs	0.04			blood culture	0.01						

Appendix Table 4. Maternal exposure and neonatal outcome definitions and prevalences (continued)

Author	Year	Maternal lab confirmed infection		Maternal clinical signs		Maternal colonization		Neonatal lab confirmed sepsis		Neonatal clinical signs of sepsis		Neonatal colonization		Neonatal infection or clincal signs	
		Definition	Prev	Definition	Prev	Definition	Prev	Definition	Prev	Definition	Prev	Definition	Prev	Definition	Prev
Niduvaje	2006					GBS vagina culture	0.02	GBS blood, csf culture	0.00						
Nolla-Salas	1998														
Orrett	2003					GBS vagina and rectum culture	0.33	GBS blood culture	0.01						
Papantoniou	1997	amniotic fluid culture or fetal blood culture	0.62												
Pass	1982														
Pearson	1967														
Persson	1986														
Philip	1982							blood culture	0.00						
Pinter	2009					S. aureus vagina culture	0.04					S. aureus nose, skin culture	0.10		
Piper	1999					GBS vagina culture	0.12	blood, csf culture	0.01						
Puchner	1993	amniotic fluid culture	0.10	chorio	0.15									blood culture or clinical signs	0.10
Puopolo	2011							blood culture	0.00						
Pylipow	1994					GBS vagina and rectum culture	0.16	blood culture	0.01						
Quentin	1989														

Appendix Table 4. Maternal exposure and neonatal outcome definitions and prevalences (continued)

Author	Year	Maternal lab confirmed infection		Maternal clinical signs		Maternal colonization		Neonatal lab confirmed sepsis		Neonatal clinical signs of sepsis		Neonatal colonization		Neonatal infection or clincal signs	
		Definition	Prev	Definition	Prev	Definition	Prev	Definition	Prev	Definition	Prev	Definition	Prev	Definition	Prev
Regan	1996					GBS cervicovagina culture	0.21	GBS blood, csf culture	0.00						
Reid	1975					GBS vagina culture	0.05			clinical signs	0.00	GBS eye, ear, throat, nose, umbilicus culture	0.00		
Rosemond	1995			chorioamnionitis	0.15			blood, csf culture	0.06						
Saez-Llorens	1995					vagina and rectum culture	0.07	blood culture	0.03						
Sensini	1997					GBS vagina culture	0.11					GBS ear, pharynx, gastric aspriate culture	0.05		
Seoud	2010					GBS vagina and rectum culture	0.18					GBS ear, pharynx, rectum culture	0.07		
Simor	1990														
Smith	2009														
Sperling	1987							blood culture	0.06						
Spinnato	1987														
Suara	1994					GBS vagina or rectum culture	0.22					GBS throat, umbilicus, rectum culture	0.23		

Appendix Table 4. Maternal exposure and neonatal outcome definitions and prevalences (continued)																
**Author**	**Year**	**Maternal lab confirmed infection**		**Maternal clinical signs**		**Maternal colonization**		**Neonatal lab confirmed sepsis**		**Neonatal clinical signs of sepsis**		**Neonatal colonization**		**Neonatal infection or clincal signs**	
		**Definition**	**Prev**	**Definition**	**Prev**	**Definition**	**Prev**	**Definition**	**Prev**	**Definition**	**Prev**	**Definition**	**Prev**	**Definition**	**Prev**
Syrogiannopoulos	1990					U. urealyticum vagina culture	0.35					U. urealyticum throat, eye, female vaginal culture	0.55		
Tafari	1979							blood culture	0.36						
Towers	1990					GBS cervicovagina culture	0.15	GBS blood culture	0.02						
Tsolia	2003					GBS vagina or rectum culture	0.07					GBS ear, throat, umbilicus culture	0.02		
Tuppurainen	1989					GBS vagina latex agglutination	0.05								
Varner	1981							blood culture	0.07						
Vergani	2002			clinical risk factors	0.16										
Visconti	1985					GBS cervicovagina culture	0.08					GBS throat, umbilicus, ear culture	0.05		
Volumenie	2001					GBS vagina culture	0.14							blood culture or clinical signs	0.01
Wallace	1983	H. influenzae blood culture	0.00					H. influenzae blood culture	0.00						

**Appendix Table 4. Maternal exposure and neonatal outcome definitions and prevalences (continued)**																	
**Author**	**Year**	**Maternal lab confirmed infection**		**Maternal clinical signs**		**Maternal colonization**		**Neonatal lab confirmed sepsis**		**Neonatal clinical signs of sepsis**		**Neonatal colonization**		**Neonatal infection or clincal signs**			
		**Definition**	**Prev**	**Definition**	**Prev**	**Definition**	**Prev**	**Definition**	**Prev**	**Definition**	**Prev**	**Definition**	**Prev**	**Definition**	**Prev**		
Weintraub	1983					GBS vagina culture	0.03					GBS ear, umbilicus culture	0.02				
Wilson	1982			clinical diagnosis with fever	0.08			blood, urine, csf culture	0.07								
Wood	1981	urine culture	0.08					undefined in paper	0.00								
Yoon	2000	amniotic fluid culture	0.27														

GBS: Group B Streptococcus; CSF: Cerebrospinal fluid
