# Supplementary material for: Prevalence of early-onset neonatal infection among newborns of mothers with bacterial infection or colonization: a systematic review and meta-analysis
Source: BMC Infect Dis. 2015 Mar 7;15:118. doi: 10.1186/s12879-015-0813-3 (PMC4364328; doi:10.1186/s12879-015-0813-3)
Supplement: Additional file 5: Figure S1. — Risk of bias summary. [file 12879_2015_813_MOESM5_ESM.pdf]

Figure 1. Risk of bias summary

Study	Loss to follow-up/not evaluated/missing data (attrition bias)	Method of handling missing data (attrition bias)	Exposed and non-exposed from the same population (selection bias)	Blinding of participants and personnel to the exposures (performance bias)	Blinding of outcome assessment to the exposures (detection bias)	Misclassification or measurement errors of exposure (information bias)	Misclassification or measurement errors of outcome (information bias)	Addressing potential confounders (confounding bias)	Other bias	Overall rating
AbeleHorn 1997	?		−		−	+	+	−	−	−
Adriaanse 1995	+		+	−	+	+	+		+	+
Andrews 2008	+		−	?	?	+	+		+	−
Averbuch 1995	?		−	−	?	+	+	−	+	−
Ayata 1994	?		?	+	+	+	+	?	+	?
Ayengar 1991	?		?	?	−	+	+		−	?
Barcaite 2009	?		?	?	?	+	+		−	?
Berardi 2011	−	−	?	−	?	+	+		−	?
Blott 1988	?		?	−	−	+	?	−	+	?
Bobitt 1977	?		?	?	?	+	−	?	+	−
Bobitt 1985	−	−	?	+	+	+	−	−	−	−
Bourgeois-Nicolaos 2010	?		?	−	−	+	+	−	−	?
Boyer 1981	−		?	−	−	+	?	?	+	?
Broekhuizen 1985	?	?	?	−	−	+	−		−	−
Buckler 2010	?			−	−	?	+		−	?
Buhimschi 2007	?		−	+	+	+	+	+	+	−
Burman 1992	?		?	?	?	+	+	?	−	?
Canpolat 2011	?		?	−	−	−	+		−	−
Cararach 1998	?					−	+	?	?	−
Carlan 1997	?					−	−		−	−
Christensen 1982	?		?	+	+	+	−	?	−	−
Christmas 1992	?		?	−	−	−	−	−	−	−
Coultrip 1994	?		?	?	?	+	−	?	−	−
Craig 1996	?		?	−	?	+	+		−	?
Cukrowska 1999	?		?	?	?	−	+	?	−	−
Cutland 2009	+	?	?	+	+	+	+	?	−	?
de Araujo 1994	?		?	?	?	−	−	−	+	−
Dollner 2002	?		?	+	+	+	−	+	−	−
Dudley 1991	?		?	?	?	+	+	?	−	?
Dutta 2010	?		?	?	?	−	+	+	−	−
Easmon 1985	−	−	?	?	?	+	+		−	?
Elder 1971	?		?	?	?	+	−	?	−	−
El-Kersh 2002	−		−	?	?	+	+	−	+	−

Figure 1. Risk of bias summary (continued)

	Loss to follow-up/not evaluated/missing data (attrition bias)	Method of handling missing data (attrition bias)	Exposed and non-exposed from the same population (selection bias)	Blinding of participants and personnel to the exposures (performance bias)	Blinding of outcome assessment to the exposures (detection bias)	Misclassification or measurement errors of exposure (information bias)	Misclassification or measurement errors of outcome (information bias)	Addressing potential confounders (confounding bias)	Other bias	Overall rating
Elzbieta 2009	?		?	?	+	+	+	?	+	?
Eren 2005	?		−	?	+	+	+	−	+	−
Faro 2010	−	−	+	−	−	+	+	−	−	+
Franciosi 1973	−	−	?	+	+	+	+	?	+	?
Frederiksen 1992	?		?	?	?	+	+	?	+	?
Gerard 1979	?		−	−	−	+	+	−	−	−
Ghanim 2011	−		−	−	−	+	?		−	−
Gibbs 1981	?		?	−	−	+	−		+	−
Gibbs 1988	+			−	+	+	+		−	+
Gilbert 2005	+	?		−	?	−	+	?	+	−
Goffinet 2005	?		?	?	?	+	−	?	+	−
Goldenberg 2008	?		?	?	?	+	+	+	+	?
Gomez 1998	?		?	−	−	+	−	+	−	−
Graham 1982	?			−	−	−	+		+	−
Hashavya 2011	?		?	−	−	+	+	?	−	?
Hervas 1993	?		?	?	?	+	+		+	?
Hickman 1999	?		+	+	+	+	+	−	+	+
Höckel 1992	?			−	+	−	+	−	+	−
Itakura 1996	?		+	?	?	+	−	−	+	−
Kadanali 2005	?		−	−	−	+	+	−	−	−
Kafetzis 2004	?		−	?	?	+	+	−	−	−
Kalinka 2006	+		?	?	?	+	−	?	+	−
Kappy 1979	?		?	?	?	+	+		−	?
Kasper 2010	+		+	?	?	+	−	?	+	−
Kishore 1987	?		?	+	+	+	+	?	+	?
Koh 1979			?	−	−	−	?		+	−
Kollee 1989	?		?	?	?	+	+	?	−	?
Kordek 2006	?			−	−	−	−	?	−	−
Kordek 2011	?		?	−	−	−	+	?	+	−
Kunze 2006	?		?	−	+	+	+	?	−	?
Kunze 2011	?		?	−	?	+	+	?	+	−

Figure 1. Risk of bias summary (continued)

	Loss to follow-up/not evaluated/missing data (attrition bias)	Method of handling missing data (attrition bias)	Exposed and non-exposed from the same population (selection bias)	Blinding of participants and personnel to the exposures (performance bias)	Blinding of outcome assessment to the exposures (detection bias)	Misclassification or measurement errors of exposure (information bias)	Misclassification or measurement errors of outcome (information bias)	Addressing potential confounders (confounding bias)	Other bias	Overall rating
Liang 1986	?		−	−	−	+	+	?	+	−
Lijoi 2007	?		?	−	?	+	+	+	−	?
Lim 1997	?		?	?	?	+	+		+	?
Matorras 1991	?		?	−	−	+	+		+	?
Matsubara 2002	?		+	?	?	+	?		+	?
Matsuda 1995	?		?	?	?	+	+	?	−	?
McCaul 1992	−		?	−	−	−	?		−	−
McGrady 1985			−	−	−	?	?	+	−	−
McLauchlin 1990			?	?	?	+	+	?	−	?
Mercer 1997	−		?	?	?	+	+	?	−	?
Mercer 1999	?		?	?	?	−	+	?	−	−
Merenstein 1980	?		?		+	+	+	?	+	?
Mitra 1997	?		?	−	+	+	+		−	?
Mitsuda 1996	?		?	?	?	+	+	?	+	?
Morales 1986	?		?	−	+	+	+		−	?
Morales 1987	?		?	−	?	+	+		−	?
Morales 1989	?		?	?	?	+	+	?	−	?
Muthusami 2007	?		+	?	?	+	+	?	+	+
Nadisauskiene 1996	?		+	+	+	−	?	?	−	−
Namavar 2008	−		−	−	+	+	+	−	−	−
Natale 1995	+	−	?	?	?	+	+	−	+	?
Newton 1989	+	−	−	−	?	−	+	?	−	−
Niduvaje 2006	?		?	−	−	+	+		−	?
Nolla-Salas 1998			?	?	?	+	+	?	+	?
Nomura 2005	−		−	?	?	+	+	+	−	−
Oddie 2002	?		?	−	−	−	+	+	−	−
Orrett 2003	?		−	−	−	+	+	?	−	−
Papantoniou 1997	−	−	?	?	+	+	+	?	−	?
Pass 1982	?		?	?	?	+	?	?	−	?
Persson 1986	?		?	?	?	+	+	?	+	?
Philip 1982	?		?	−	+	−	+		+	−
Pinter 2009	−	−	?	?	?	+	+	?	+	?

Figure 1. Risk of bias summary (continued)

	Loss to follow-up/not evaluated/missing data (attrition bias)	Method of handling missing data (attrition bias)	Exposed and non-exposed from the same population (selection bias)	Binding of participants and personnel to the exposures (performance bias)	Binding of outcome assessment to the exposures (detection bias)	Misclassification or measurement errors of exposure (information bias)	Misclassification or measurement errors of outcome (information bias)	Addressing potential confounders (confounding bias)	Other bias	Overall rating
Piper 1999	?		?	?	?	+	+	+	−	?
Puchner 1993	?		?	?	?	−	−		−	−
Puopolo 2011	?		−			+	+	+	+	−
Pylipow 1994	?		?	−	−	+	+	?	−	?
Quentin 1989				−	−	+	+		+	+
Regan 1996	−	−	−	−	?	+	+	?	−	−
Reid 1975	?		?	−	−	+	+		−	?
Ronnestad 2005	?		?	?	?	−	+	+	−	−
Rosemond 1995	?		?	−	−	+	+	?	−	?
Saez-Llorens 1995	+	−	?	?	+	+	+		−	?
Sensini 1997	?		?	?	+	+	+	?	−	?
Seoud 2010	−	−	+	?	?	+	+	−	−	+
Simor 1990	?		?	−	−	+	−		+	−
Smith 2009	−			−	−	+	+		+	+
Sperling 1987	?			−	−	−	+		−	−
Spinnato 1987	?			−	?	−	?		−	−
Suara 1994	−	−	−	?	?	+	+	?	+	−
Syrogiannopoulos 1990	+		?	−	−	+	+		+	?
Tafari 1979	?		?	?	?	−	+	?	−	−
Tameliene 2010	?		?	?	?	+	+	+	+	?
Towers 1990	?		?	?	?	+	?	?	−	?
Tsolia 2003	−		−	?	?	+	+	−	−	−
Tuppurainen 1989	?		?	−	−	+	+	?	+	?
Varner 1981	?			−	−	−	+		−	−
Vergani 2002	?		?	−	−	+	+	?	−	?
Visconti 1985	+		?	?	+	+	+	?	+	?
Volumenie 2001	−		?	−	−	+	−	?	−	−
Wallace 1983				−	−	+	+		+	+
Weintraub 1983	?		?	?	+	+	+	?	+	?
Wilson 1982			?	−	−	−	+	?	−	−
Wood 1981	?		?	−	−	+	+		−	?
Yoon 2000	?		+	?	?	−	+	+	+	−
